# Associations of suffering with facets of health and well-being among working adults: longitudinal evidence from two samples

**DOI:** 10.1038/s41598-022-24497-8

**Published:** 2022-11-22

**Authors:** Richard G. Cowden, Andrew J. Seidman, Charlotte Duffee, Dorota Węziak-Białowolska, Eileen McNeely, Tyler J. VanderWeele

**Affiliations:** 1 Human Flourishing Program, Harvard University, Cambridge, MA USA; 2 Department of Psychiatry, University of Pittsburgh, Pittsburgh, PA USA; 3 Sustainability and Health Initiative (SHINE), Department of Environmental Health, Harvard T. H. Chan School of Public Health, Boston, MA USA; 4 Centre for Evaluation and Analysis of Public Policies, Faculty of Philosophy, Jagiellonian University, Kraków, Poland; 5 Department of Epidemiology, Harvard T. H. Chan School of Public Health, Boston, MA USA

**Keywords:** Psychology, Signs and symptoms

## Abstract

Suffering is an experiential state that every person encounters at one time or another, yet little is known about suffering and its consequences for the health and well-being of nonclinical adult populations. In a pair of longitudinal studies, we used two waves of data from garment factory workers (Study 1 [T1: 2017, T2: 2019]: *n* = 344) and flight attendants (Study 2 [T1: 2017/2018, T2: 2020]: *n* = 1402) to examine the prospective associations of suffering with 16 outcomes across different domains of health and well-being: physical health, health behavior, mental health, psychological well-being, character strengths, and social well-being. The primary analysis involved a series of regression analyses in which each T2 outcome was regressed on overall suffering assessed at T1, adjusting for relevant sociodemographic characteristics and the baseline value (or close proxy) of the outcome assessed at T1. In Study 1, associations of overall suffering with worse subsequent health and well-being were limited to a single outcome on each of the domains of physical health and mental health. Overall suffering was more consistently related to worse subsequent health and well-being in Study 2, with associations emerging for all but two outcomes. The pattern of findings for each study was largely similar when aspects of suffering were modeled individually, although associations for some aspects of suffering differed from those that emerged for overall suffering. Our findings suggest that suffering may have important implications for the health and well-being of worker populations.

## Introduction

Scholars within various disciplines, including the humanities, medicine, bioethics, and social sciences, have long been interested in suffering and its consequences for human well-being^1–3^. Prominent existential psychologist, Viktor Frankl, described suffering as “an ineradicable part of life” without which “human life cannot be complete” (p. 76)^4^. Similarly, physician Eric Cassell, a pioneer in the medical literature on suffering, regarded the existence of suffering as a ‘universal’ element of human life in every culture^5^. Not only does this imply that suffering “is entangled in the very essence of human existence” (p. 48)^6^, but it also suggests that suffering is not isolated to certain populations (e.g., people with mental health problems, victims of tragedy). Thus, suffering has potential implications for the well-being of all persons, and is important for both understanding and improving public health^7–9^.

Although suffering is a ubiquitous part of the human experience, the extant empirical literature on suffering is characterized by a disproportionate focus on Western samples and older adults living with physical illness^10,11^. Such evidence has provided some insight into the antecedents, experiences, and consequences of suffering that are relevant to clinical populations of adults who are dealing with physical health symptoms and illness, but knowledge of these dynamics among healthy adults and people living in non-Western contexts is more limited. Indeed, only recently has empirical research begun to show that suffering has relevance among nonclinical populations living both within and beyond the traditional boundaries of the West^12,13^.

In addition, empirical research on suffering has almost exclusively been cross-sectional. This body of literature has contributed substantially to knowledge about suffering as it is experienced contemporaneously with issues or ailments in the domain of physical health. However, most cross-sectional studies cannot be used to draw causal inferences, which is necessary to distinguish more clearly between the causes and consequences of suffering and identify the most appropriate avenues for addressing suffering in different populations. For example, cross-sectional data may suggest that suffering is associated with a major depressive episode, but that might be because those who become depressed are more likely to experience suffering or because suffering itself may lead to depression. Ambiguity concerning the temporal ordering of associations with suffering could undermine or limit the effectiveness of treatments and interventions that aim to support those who are suffering. To build on the foundational quantitative work that has explored suffering and its relations with facets of well-being, more rigorous longitudinal studies are needed in a wider range of populations and contexts.

One setting in which suffering appears to have relevance is work. Previous studies have found that suffering is associated with lower well-being in a variety of life domains^2,13^, and evidence from studies involving workers suggest that lower well-being in life can negatively affect well-being at work and job performance^14,15^. The potential impacts of suffering on an employee’s well-being and performance could have downstream consequences on the organization more broadly (e.g., lower profitability), which may further jeopardize the livelihood of employees who are suffering (e.g., potential termination of employment). Although “silence about suffering at work” (p. 8)^16^ represents an obstacle to promoting occupational health and maximizing work performance, organizations are also uniquely positioned to support the needs of their employees because of how much time employees typically spend engaged with the organizations for which they work. For example, the average adult spends up to half of their daily waking hours at work^17^, and most working adults spend more time during an average week engaged in work than any other activity besides sleep^18^. Thus, an expanded literature on suffering and its implications for the well-being of workers could provide organizations with insights that inform how they can strengthen their commitment to promoting worker well-being and further cultivate a ‘caring climate’ by supporting employees who are suffering. In the present set of studies, we use longitudinal data from samples of healthy workers employed in Western and non-Western contexts to examine the associations between suffering and a variety of subsequent health and well-being outcomes.

### Suffering, health, and well-being

Suffering might be understood as an undesired experiential state, of considerable duration or intensity, involving the loss or privation of some perceived good^8,13^. Although suffering has a negative affective quality that can be challenging to differentiate from symptoms or conditions laden with negative affect (e.g., pain, depressed mood), a person is only thought to be suffering when their negative affective experience is accompanied by an “occurrent desire that it not be occurring” (p. 27)^19^. This suggests that a person may be in severe pain or meet diagnostic criteria for depression but not qualify as suffering. Such distinctions have been demonstrated empirically. For example, Body et al.^20^ found that nearly one fifth of emergency department patients who reported a high level of pain indicated they were not suffering. Similarly, Cowden et al.^2^ showed that suffering is associated with lower subsequent psychological flourishing even after adjusting for (amongst other factors) symptoms of anxiety and depression. These findings indicate that suffering is a distinct negative affective experience, and that its presence could have clinically significant implications for individual well-being^12,21^.

Suffering is a multifaceted experience involving mind–body processes that intersect psychological, physical, spiritual, and sociocultural dimensions^22^. Although the cause of suffering may be isolated to a particular domain of functioning (e.g., loss of employment, death of a loved one), the experience of suffering itself is often not constrained to a single dimension of a person’s life and will usually permeate various (if not all) aspects of one’s life^23^. As such, suffering is without boundaries; it has a pervasive quality that can be characterized as an experience that involves the *whole person*^8,24^. On this integrative view, the diffuse and permeating nature of suffering has the potential to impact many different facets of health and well-being^13^.

Much of the existing literature that has reported on the associations between first-person experiences of suffering and well-being focuses on facets within the domain of physical health. Given the tendency for empirical research on suffering to emphasize clinical populations dealing with physical health problems or illness, it is not surprising that numerous (mostly cross-sectional) studies have found evidence to suggest that suffering is associated with worse physical health and/or functioning^10,25,26^. Many (mostly cross-sectional) studies have also intersected the domains of mental health (e.g., anxiety symptoms, depression symptoms) and/or psychological well-being (e.g., happiness, meaning in life), with findings largely pointing to higher psychological distress and lower psychological well-being among those who are suffering^27–29^.

Relatively few studies have addressed other domains of well-being, including character strengths and social well-being; these are also the domains for which evidence appears to be more mixed. For example, some cross-sectional studies have shown that suffering is associated with higher scores on indices of strengthened character (e.g., perceived posttraumatic growth)^30^ and lower scores on certain facets of social well-being (e.g., heightened social isolation)^29^. However, one recent longitudinal study found little evidence to suggest that suffering predicted changes in selected character strengths (e.g., being oriented toward promoting good) and comparable facets of social well-being (e.g., satisfaction with relationships) approximately 1 month later^13^, though of course such changes may take more than 1 month to occur. Although the current literature suggests that suffering is generally related to worse well-being, the available evidence is characterized by an overreliance on cross-sectional data among physically ill older adults and a disproportionate emphasis on facets of well-being within certain domains (e.g., physical health). Hence, more robust evidence is needed to develop an improved understanding of the relationship between suffering and different facets of health and well-being.

### The present research

To address some of the gaps in knowledge and enrich the existing body of literature on suffering, we use longitudinal data from two worker samples (Study 1: garment factory workers; Study 2: flight attendants) to examine the associations between an index of overall suffering and a wide range of subsequent health and well-being outcomes. Both studies follow the analytic template for outcome-wide longitudinal designs^31^, which provides a framework for addressing potential confounding and reverse causation to develop a more complete picture and integrative understanding of how suffering might affect well-being across numerous outcomes. To complement our primary analysis with overall suffering as the exposure, we also explore the effect that each specific aspect of suffering (e.g., intensity of suffering, duration of suffering, perceived powerlessness over suffering) might have on the health and well-being outcomes. Such evidence could help organizational stakeholders and practitioners make more informed decisions about interventions into suffering, particularly those (e.g., management, occupational health psychologists) who are often tasked with identifying cost-effective strategies for promoting employee well-being across the organization.

## Study 1

In Study 1, we examined the longitudinal associations of suffering with different facets of health and well-being in a sample of garment factory workers from Sri Lanka. Garment factory work in South and Southeast Asia is a precarious, physically demanding form of employment characterized by a lack of job security, unsafe or unhealthy working environments, insufficient compensation, and long working hours^32^. Although we are not aware of research that has explicitly reported on experiences of suffering among people engaged in precarious work, evidence suggests that precarious employment is associated with higher perceived stress that can have downstream consequences on mental and physical well-being^33–35^. It is possible that precarious employment and its impact on a person’s general quality of life might precipitate suffering, which could degrade well-being over time. To explore this theorizing, we used data from a sample of Sri Lankan garment factory workers to estimate the effects of overall suffering and each of its aspects on 16 physical health, health behavior, mental health, psychological well-being, character strengths, and social well-being outcomes assessed approximately 2 years later. Drawing on prior research, we expected that suffering and each of its aspects would generally be associated with worse subsequent health and well-being.

### Methods

#### Study sample

Data for this study were taken from the Sri Lankan cohort of the Worker Well-Being Survey (WWBS), a multi-country research project developed to assess and monitor the well-being of apparel industry workers within the supply chains of international clothing brands. Details about the aims, scope, and methodology of the WWBS can be found elsewhere^36^. Our study focuses exclusively on the Sri Lankan sample because suffering was only assessed in that country.

The baseline (T1) survey was completed in August 2017, which is when participants completed a measure of suffering. With permission from management, employees of several garment factories in South and Central Sri Lanka were invited to participate in the study. Prospective participants were provided background information and instructions about how to complete the T1 survey. After providing electronic informed consent, participants responded to the self-administered survey via a secure electronic tablet application. A similar procedure was followed when those who were still employed and had responded to the T1 survey were invited to complete a follow-up survey (T2) approximately 2 years later in November 2019. For both waves, participants who responded to the survey were entered into a lottery draw to win one of several prizes (e.g., clothing, food items).

Participants were given the option of completing the survey in English, Sinhala, or Tamil. To ensure that the survey items were culturally and linguistically appropriate for the apparel industry worker population in Sri Lanka, an iterative, multiphased translation procedure was used to translate the survey items to Sinhala and Tamil. A comprehensive description of the approach that was used to translate the survey items to these local languages can be found in Węziak-Białowolska et al.^36^. Both T1 and T2 surveys contained items addressing a wide range of topical areas, including workplace experiences, health at work, and well-being more generally, but there were some differences between the survey items that were administered in the two waves.

We identified 1258 individuals who responded at T1. Of those, *n* = 344 (27.34%) responded to the T2 survey. Independent samples *t*-tests and Chi-square tests of independence were used to compare the baseline characteristics of participants who remained in the cohort to those lost to follow-up (see Supplemental Table S1). Participants who were older, female, employed for more than 5 years, currently smoking, reported less days with pain-related limitations, and had fewer sleepless days were more likely to be retained over time (*p*s ≤ 0.049).

Table 1 describes the baseline sociodemographic characteristics of the participants who responded at both T1 and T2 (*n* = 344). Participants (*M*_age_ = 31.71, *SD* = 9.04) were predominantly female (65.41%) and self-identified ethnically as Sinhalese (90.96%). Nearly two-thirds of the participants were married (60.17%), and slightly more than half reported having child dependents (50.48%) or adult dependents (51.90%). A majority of the sample had completed some postsecondary education or higher (56.40%), and most had been employed in their role for 5 years or less (70.64%).

**Table 1:** Distribution of participant characteristics in Study 1 (factory worker sample) and Study 2 (flight attendant sample).

Characteristic	Study 1: Factory worker sample (*n* = 344)		Study 2: Flight attendant sample (*n* = 1402)	
	*n* (%)	*M* ± *SD* (range)	*n* (%)	*M* ± *SD* (range)
**Age (years)**	344	31.71 ± 9.04 (18–55)	1398	56.09 ± 6.10 (46–75)
18–24 years	88 (25.58)		0 (0.00)	
25–34 years	128 (37.21)		0 (0.00)	
35–44 years	95 (27.62)		0 (0.00)	
≥ 45 years	33 (9.59)		1398 (100.00)	
**Gender**	344		1402	
Female	225 (65.41)		1155 (82.38)	
Male	119 (34.59)		247 (17.62)	
**Sexual orientation**	–		1398	
Heterosexual	–		1238 (88.56)	
Other	–		160 (11.44)	
**Racial status**	–		1399	
American Indian and Alaska Native	–		4 (0.29)	
Asian	–		50 (3.57)	
Black or African American	–		48 (3.43)	
Native Hawaiian and Other Pacific Islander	–		1 (0.07)	
White	–		1181 (84.42)	
Two or more races	–		50 (3.57)	
Other	–		65 (4.65)	
**Ethnic status**	332		–	
Indian Lankan Tamil	8 (2.41)		–	
Sinhalese	302 (90.96)		–	
Sri Lankan Tamil	18 (5.42)		–	
Two or more ethnicities	4 (1.20)		–	
**Marital status**	344		1238	
Single	91 (26.45)		188 (15.19)	
Married	207 (60.17)		654 (52.83)	
Living with partner	22 (6.40)		112 (9.05)	
Separated	11 (3.20)		20 (1.62)	
Divorced	6 (1.74)		245 (19.79)	
Widowed	7 (2.03)		19 (1.53)	
**Educational attainment**	328		1239	
Up to high school equivalency	143 (43.60)		0 (0.00)	
Up to completion of undergraduate degree	184 (56.10)		1094 (88.30)	
Graduate school/advanced degree	1 (0.30)		145 (11.70)	
**Child dependents**	311		1205	
None	154 (49.52)		991 (82.24)	
One	64 (20.58)		127 (10.54)	
Two	68 (21.86)		70 (5.81)	
≥ Three	25 (8.04)		17 (1.41)	
**Older adult dependents**	316		1234	
None	152 (48.10)		1053 (85.33)	
One	147 (46.52)		126 (10.21)	
Two	6 (1.90)		38 (3.08)	
≥ Three	11 (3.48)		17 (1.38)	
**Employment tenure**	344		1396	
Up to 1 year	89 (25.87)		3 (0.21)	
1–5 years	154 (44.77)		1334 (95.56)	
> 5 years	101 (29.36)		59 (4.23)	

*M* mean, *SD* standard deviation.

#### Measures

##### Exposure

Participants completed the Personal Suffering Assessment (PSA)^8^ at T1. The PSA is a seven-item measure that assesses a person’s general experience of suffering at present. The first item is a global question that asks about the extent to which a person is suffering; the remaining six items capture different aspects of a person’s experience of suffering (i.e., intensity, duration, powerlessness, pervasiveness, disruption to purposes, threats to personhood). An 11-point response scale (from 0 to 10) is used to rate each item (e.g., “To what extent are you suffering?”), with orienting labels presented alongside anchor points at each end of the scale (see Supplemental Table S2). Consistent with previous studies, an overall suffering score was calculated by averaging responses to all seven items (α = 0.88). We also modelled each of the items individually to obtain a more fine-grained understanding of how different aspects of suffering might be associated with the outcomes of interest.

##### Outcomes

Applying a multidimensional conception of health and well-being^37–39^, we examined 16 health and well-being outcomes that were assessed at T2 via well-validated and widely used measures (see Supplemental Table S2). Outcomes included indices of physical health (i.e., general health, physical health, pain-related limitations, disability days), health behavior (i.e., adequate sleep, current smoking), mental health (i.e., mental health, depressed mood), psychological well-being (i.e., life satisfaction, happiness, meaning in life, sense of purpose), character strengths (i.e., promote good, delay gratification), and social well-being (i.e., satisfying relationships, content relationships). Additional information about the measurement of each outcome is provided in Supplemental Table S2.

##### Covariates

We adjusted for several sociodemographic covariates assessed at T1, including age (continuous), gender (female vs. male), ethnic status (Sinhalese vs. other), marital status (married vs. other), child dependents (no vs. yes), older adult dependents (no vs. yes), educational attainment (up to high school equivalency vs. some postsecondary education or higher), and employment tenure (≤ 5 years vs. > 5 years). When available, we also adjusted for the prior value of the respective outcome variable assessed at T1. Three outcomes (i.e., pain-related limitations, adequate sleep, depressed mood) were not assessed at T1; therefore, models for each of these outcomes adjusted for a conceptually equivalent variable that was assessed at T1. We provide details about which T1 variables were used as close proxies for each of these outcomes in Supplemental Table S2, with additional information included in other tables when results are presented for multivariate models that make use of these proxies.

#### Ethics approval

The WWBS was granted institutional ethical approval from the Harvard Longwood Campus Institutional Review Board. All procedures involving human participants were performed in accordance with the 1964 Helsinki declaration and its later amendments.

#### Informed consent

Informed consent was obtained from all individual participants included in this study.

### Results and discussion

All statistical analyses were performed in R^40^. We began by computing a preliminary set of analyses using an available-case approach. Descriptive statistics for all T1 and T2 study variables in the analytic sample can be found in Supplemental Table S3. The mean for overall suffering was 3.96 (*SD* = 2.86). Means for intensity of suffering (*M* = 4.34, *SD* = 3.81), length of suffering (*M* = 4.16, *SD* = 3.76), powerlessness over suffering (*M* = 4.24, *SD* = 3.95), pervasiveness of suffering (*M* = 4.41, *SD* = 4.01), and disruption to purposes (*M* = 4.37, *SD* = 3.80) were higher than the mean for overall suffering, whereas means were lower for extent of suffering (*M* = 3.01, *SD* = 3.10) and threats to personhood (*M* = 3.79, *SD* = 3.72). Correlations between the seven aspects of suffering ranged from *r* = 0.26 to 0.81 (see Supplemental Table S4).

We examined cross-sectional and prospective correlations of overall suffering assessed at T1 with each outcome assessed at T1 and T2, respectively (see Supplemental Table S5). Effect sizes are interpreted using guidelines offered by Funder and Ozer^41^. Overall suffering was cross-sectionally correlated (small to large in magnitude) with worse health and well-being on three indices of physical health (i.e., general health, pain-related limitations days, disability days), both indices of mental health (i.e., mental health, depressed mood days), two indices of psychological well-being (i.e., meaning in life, sense of purpose), and the character strength of being oriented toward promoting good (*r*s = |0.15 to 0.29|). In contrast, overall suffering was prospectively correlated (medium in magnitude) with worse health and well-being on two outcomes, namely pain-related limitations and depressed mood (*r*s = 0.17 and 0.18, respectively). Prospective correlations tended to be smaller in magnitude compared to cross-sectional correlations.

#### Primary analysis: associations of overall suffering with health and well-being outcomes

Missing data were imputed on all study variables using multiple imputation by chained equations^42^. We generated 10 datasets, performed analyses using each imputed dataset, and then combined the results across imputations^43^. Following the analytic template for the outcome-wide longitudinal design^31^, we estimated a series of models in which each of the 16 outcome variables at T2 was regressed on overall suffering assessed at T1 (one outcome at a time). For the 14 continuous outcome variables, multiple linear regression models were estimated. A logistic regression model was estimated for the outcome of current smoking (prevalence of < 10%), and a generalized linear model with a log link and Poisson distribution was estimated for the outcome of adequate sleep (prevalence of ≥ 10%). All analyses adjusted for the full set of sociodemographic characteristics and the baseline value (or close proxy) of the respective outcome assessed at T1. To facilitate comparison of effect estimates across continuous outcomes, we standardized all continuous outcomes (*M* = 0, *SD* = 1). We applied Bonferroni corrections to account for multiple testing, but we focus our interpretation on results before Bonferroni correction because practices for multiple testing vary and continue to evolve^44–46^. However, to acknowledge the different practices and cutoffs that can be applied, all tables that include the results of multivariable analyses report *p*-values both before and after Bonferroni correction.

Results for the estimated effects of overall suffering assessed at T1 on each of the subsequent outcomes assessed at T2 are reported in Table 2. Overall suffering evidenced positive associations (medium in magnitude) with subsequent pain-related limitations (β = 0.20, *p* < 0.001) and depressed mood (β = 0.16, *p* = 0.006). There was little evidence to suggest that overall suffering was associated with any of the other 14 outcomes (*p*s > 0.05). Results were largely comparable when the analyses were replicated with overall suffering modeled as a categorical variable using tertiles, with tertile 1 serving as the reference category (see Supplemental Table S6). Similar results also emerged when the analyses were replicated using complete cases (see Supplemental Table S7), except that overall suffering was no longer associated with depressed mood.

**Table 2:** Associations of overall suffering (T1) with health and well-being outcomes (T2) in Study 1 (factory worker sample).

Outcome	Effect estimate		*E*-values^a^	
	Reference	β/OR/RR [95% CI]	Effect estimate^b^	CI limit^c^
**Physical health**				
General health	0.00	− 0.11 [− 0.22, 0.00]	1.45	1.00
Physical health	0.00	− 0.04 [− 0.16, 0.08]	1.23	1.00
Pain-related limitations^#^	0.00	0.20 [0.09, 0.32]***	1.69	1.39
Disability days	0.00	0.06 [− 0.06, 0.19]	1.31	1.00
**Health behavior**				
Adequate sleep^##^	1.00	1.00 [0.91, 1.09]	1.04	1.00
Current smoking	1.00	0.65 [0.32, 1.32]	2.44	1.00
**Mental health**				
Mental health	0.00	− 0.06 [− 0.17, 0.05]	1.31	1.00
Depressed mood^###^	0.00	0.16 [0.05, 0.28]*	1.58	1.26
**Psychological well-being**				
Life satisfaction	0.00	0.02 [− 0.09, 0.13]	1.15	1.00
Happiness	0.00	− 0.01 [− 0.12, 0.10]	1.13	1.00
Meaning in life	0.00	− 0.02 [− 0.14, 0.09]	1.17	1.00
Sense of purpose	0.00	− 0.00 [− 0.12, 0.12]	1.06	1.00
**Character strengths**				
Promote good	0.00	0.04 [− 0.08, 0.15]	1.22	1.00
Delay gratification	0.00	− 0.04 [− 0.15, 0.07]	1.24	1.00
**Social well-being**				
Satisfying relationships	0.00	0.04 [− 0.08, 0.16]	1.25	1.00
Content relationships	0.00	0.02 [− 0.08, 0.13]	1.17	1.00

*β* standardized effect size, *CI* confidence interval, *OR* odds ratio, *RR* risk ratio. *n* = 344 for all analyses. Multiple imputation was performed to impute missing data on the exposure, covariates, and outcomes. We ran a different type of model depending on the nature of the outcome: (1) for each continuous outcome, we ran a linear regression model to estimate a β; (2) for the outcome of current smoking (prevalence of < 10%), we ran a logistic regression model to estimate an OR; (3) for the outcome of adequate sleep (prevalence of ≥ 10%), we ran a generalized linear model with a log link and Poisson distribution to estimate a RR. If the reference value is “0,” the effect estimate is β; if the reference value is “1,” the effect estimate is OR or RR. Each continuous outcome was standardized (*M* = 0, *SD* = 1). All models adjusted for prior values of age, gender, ethnic status, marital status, educational attainment, job tenure, child dependents, and older adult dependents assessed at T1. Unless otherwise indicated, all models also adjusted for the prior value of the respective outcome assessed at T1. If a prior value of an outcome was not available, we adjusted for the prior value of a variable that was most comparable to the outcome: ^#^pain-related limitations days, ^##^sleepless days, ^###^depressed mood days (see Supplemental Table S2). *p < 0.05 before Bonferroni correction, ***p < 0.05 after Bonferroni correction (the *p*-value cutoff for Bonferroni correction was 0.05/16 = 0.003 for each outcome). ^a^The formula for calculating *E*-values can be found in VanderWeele and Ding^47^. ^b^*E*-values for effect estimates are the minimum strength of association that an unmeasured confounder would need to have with both the exposure and the outcome variable to fully explain away the observed effect, after accounting for the measured covariates. ^c^*E*-values for the limit of the 95% CI closest to the null denote the minimum strength of association that an unmeasured confounder would need to have with both the exposure and the outcome variable to shift the confidence interval to include the null value, after accounting for the measured covariates.

We used *E*-values to assess the robustness of the results from the primary analysis to potential unmeasured confounding^47^. *E*-values estimate the minimum strength of association that an unmeasured confounder would need to have with both the exposure and outcome (on the risk ratio scale), above and beyond adjusted covariates, to fully explain away the observed exposure-outcome association. *E*-values can range from 1.00 to any number greater than 1.00; higher values indicate that stronger unmeasured confounder risk ratio associations would be needed to explain away the association between the exposure and outcome. For the associations that were observed in the primary analysis, *E*-values ranged from 1.06 to 2.44 for the effect estimates and 1.00 to 1.39 for the confidence interval limits closest to the null (see Table 2). *E*-values suggested that the observed associations for pain*-*related limitations (effect estimate: 1.69, confidence interval limit: 1.39) and depressed mood (effect estimate: 1.58, confidence interval limit: 1.26) were somewhat robust to potential unmeasured confounding.

#### Secondary analysis: associations of suffering aspects with health and well-being outcomes

We repeated the primary analysis after replacing overall suffering with each aspect of suffering as the exposure variable (one aspect of suffering and one outcome at a time). All seven aspects of suffering were modeled as continuous variables. The results for this set of analyses can be found in Supplemental Table S8. Consistent with the results of the primary analysis, each aspect of suffering was positively associated with subsequent pain-related limitations (medium in magnitude); the largest effect size emerged for pervasiveness of suffering, followed by powerlessness over suffering and then disruption to purposes. Powerlessness over suffering and pervasiveness of suffering were also positively associated with depressed mood (medium in magnitude). Length of suffering and threats to personhood were negatively associated with general health, whereas threats to personhood was positively associated with satisfying relationships (medium in magnitude). There was little evidence to suggest that the aspects of suffering were associated with other health and well-being outcomes (*p*s > 0.05).

### Summary

In this study of garment industry factory workers, the findings provided modest evidence to suggest that overall suffering is associated with subsequently greater pain-related limitations and an increase in depressed mood approximately 2 years later. The pattern of associations for the aspects of suffering was mostly consistent with the findings for overall suffering, with some heterogeneity. For example, all seven aspects of suffering were associated with pain-related limitations, but only two aspects of suffering were associated with depressed mood. In addition, certain aspects of suffering were associated with outcomes for which there was little evidence of association with overall suffering. To illustrate, overall suffering was unrelated to general health, whereas length of suffering was associated with a decrease in subsequent general health. In general, however, we found limited evidence that suffering is associated with worse health and well-being.

## Study 2

In Study 2, we examined the associations between suffering experienced by flight attendants prior to the coronavirus disease 2019 (COVID-19) pandemic and various health and well-being outcomes assessed during the public health crisis. Flight attendant work is a challenging form of employment characterized by a unique set of job demands. For example, flight attendants perform repetitive job tasks in a confined setting, often have non-routine sleep schedules due to irregular working hours (e.g., overnight flights), and many are exposed to disorderly or inappropriate behavior (e.g., sexual harassment) while performing their duties^48–50^. Although these kinds of adverse working conditions can be a direct source of increased risk for distress and other health-related consequences^51^, the indirect effects of flight attendant work (e.g., disrupted social rhythms by nature of routine travel and frequently being away from home) also have the potential to degrade well-being^52^.

Some evidence suggests that the job-related difficulties of flight attendant work were exacerbated by the COVID-19 pandemic^53^, in which flight attendants were required to perform their core duties while adhering to public health regulations (e.g., maintaining social distance, ensuring hygiene protocols were being followed) and enforce novel air travel rules (e.g., mask mandates) to protect the health of passengers and crew on board^54^. Flight attendants were also not exempt from the broader impacts of the COVID-19 pandemic, such as fear of severe acute respiratory syndrome coronavirus 2 infection and increased job insecurity^55^. It is possible that the confluence of work and life stressors precipitated by the COVID-19 pandemic may have had particularly devastating consequences for the well-being of flight attendants who were suffering before the public health crisis. Following the analytic approach in Study 1, we estimated the effects of overall suffering and each of its aspects assessed approximately 2 years pre-COVID-19 with 16 physical health, health behavior, mental health, psychological well-being, character strengths, and social well-being outcomes assessed a few months into the COVID-19 pandemic. Based on previous literature and the timing of the assessed outcomes, we anticipated that pre-COVID-19 overall suffering and each of its aspects would generally be associated with worse health and well-being during the COVID-19 pandemic.

### Methods

#### Study sample

This study used the third and fourth waves of data from the Flight Attendant Health Study (FAHS). The FAHS is a longitudinal health surveillance study of flight attendants, most of whom are employed by commercial airlines operating in the US, UK, and/or Canada. For more details about the aims, scope, and methodology of the FAHS, see McNeely et al.^56^.

Wave three data collection took place between July 2017 and December 2018, and it was the first and only wave in which a measure of suffering was administered. Hence, wave three was chosen as the baseline wave (T1) for this study. To strengthen inferences about causality, we used wave four as the outcome wave (T2) because data collection took place approximately 2 years after the wave in which the exposure of suffering was assessed. Although the T1 and T2 surveys were not identical, items in both surveys covered numerous topics related to health and well-being at work and life more generally.

Both current and former flight attendants who were previously enrolled in the FAHS were invited to participate at T1. The existing sample was replenished with additional flight attendants who were recruited through local and national worker unions, postcards that were sent directly to flight attendants who were employees of airlines that agreed to support the FAHS, and recruitment campaigns that took place on airport premises.

At T1, prospective participants were given an overview of the FAHS and directed to complete a web-based survey. All participants were required to provide electronic informed consent before responding to the self-administered survey in English. The same procedure was followed at T2. In both waves, those who responded to the survey were incentivized with an opportunity to enter a lottery draw to win one of 10 Amazon gift cards (valued at $99 each at T1 and $50 each at T2).

There were *N* = 10378 current and former flights attendants who responded to the T1 survey prior to the end of 2018. Based on the scope and objectives of this study, we made an a priori decision to restrict our analyses to participants who self-reported that they were currently employed as a flight attendant at T1 (*n* = 7338). Of the eligible participants who responded to the T1 survey, *n* = 1402 (19.11%) participated in the T2 survey several months into the COVID-19 pandemic (July to September 2020). We used Chi-square tests of independence and independent samples *t*-tests to compare the baseline characteristics of those who participated in the follow-up survey and those who did not (see Supplemental Table S9). Those who were older, female, heterosexual, White, married, did not have child dependents, had older adult dependents, and were employed as a flight attendant for 5 years or less were more likely to have participated at T2 (*p*s ≤ 0.009). Those who participated in both waves endorsed lower overall suffering (*p* = 0.001) and reported significantly greater health and well-being on most indices of physical health, mental health, psychological well-being, character strengths, and social well-being. However, those who did not participate in the T2 survey reported fewer days with pain-related limitations than those who participated in both waves (*p* = 0.002).

Baseline sociodemographic characteristics of the participants who participated at T1 and T2 (*n* = 1402) can be found in Table 1. Participants (*M*_age_ = 56.09, *SD* = 6.10) were predominantly female (82.38%), heterosexual (88.56%), and identified racially as White (84.42%). Approximately half of the sample was married (52.83%), and most did not have child dependents (82.24%) or older adult dependents (85.33%). Most of the sample was educated up to the completion of an undergraduate degree (88.30%), and almost all participants indicated that they had been employed as a flight attendant for 5 years or less (95.77%).

#### Measures

##### Exposure

Participants completed the seven-item measure of subjective suffering (i.e., PSA)^8^ that was administered in Study 1 (α = 0.95).

##### Outcomes

We included 16 indicators of health and well-being as outcomes at T2, which were assessed via well-validated and widely used measures (see Supplemental Table S10). Most outcomes overlapped with those that were examined in Study 1. The outcomes represented different dimensions of health and well-being, including physical health (i.e., general health, physically unhealthy days, pain-related limitations days, disability days, fatigue days, vitality days), health behavior (i.e., sleepless days), mental health (i.e., mentally unhealthy days, depressed mood days), psychological well-being (i.e., life satisfaction, happiness, meaning in life, sense of purpose), character strengths (i.e., promote good, delay gratification), and social well-being (i.e., satisfying relationships). Further details about the measurement of the outcomes, including specific item wording and operationalization of the variables, can be found in Supplemental Table S10.

##### Covariates

We adjusted for a similar set of sociodemographic characteristics to those included in Study 1, each of which was assessed at T1: age (continuous), gender (female vs. male), sexual orientation (heterosexual vs. other), racial status (White vs. other), marital status (married vs. other), child dependents (no vs. yes), older adult dependents (no vs. yes), educational attainment (up to completion of undergraduate degree vs. graduate school/advanced degree), and employment tenure (≤ 5 years vs. > 5 years). To reduce the possibility of reverse causation and further address potential confounding, we adjusted for the prior value of the respective outcome variable assessed at T1 when data were available. Both fatigue days (past 30 days) and depressed mood days were not assessed at T1. As a result, we adjusted for a conceptually equivalent variable that was included in the T1 survey. Information about T1 variables that were used as close proxies for the abovementioned outcomes can be found in Supplemental Table S10, with further details provided in other tables that contain results for multivariate models involving these proxies.

#### Ethics approval

The FAHS was granted institutional ethical approval from the Harvard Longwood Campus Institutional Review Board. All procedures involving human participants were performed in accordance with the 1964 Helsinki declaration and its later amendments.

#### Informed consent

Informed consent was obtained from all individual participants included in this study.

### Results and discussion

Our analytic strategy in Study 2 was comparable to Study 1. Using an available-case approach, we computed descriptive statistics for all T1 and T2 study variables in the analytic sample (see Supplemental Table S3). The mean for overall suffering was 2.21 (*SD* = 2.41). Means for extent of suffering (*M* = 2.84, *SD* = 2.56), pervasiveness of suffering (*M* = 2.67, *SD* = 3.19), and disruption to purposes (*M* = 2.46, *SD* = 3.02) were higher than the mean for overall suffering, whereas means were lower for intensity of suffering (*M* = 1.86, *SD* = 2.35), length of suffering (*M* = 1.91, *SD* = 2.50), powerlessness over suffering (*M* = 1.98, *SD* = 2.73), and threats to personhood (*M* = 1.80, *SD* = 2.70). Correlations between the seven aspects of suffering ranged from *r* = 0.66 to 0.87 (see Supplemental Table S4).

We also report cross-sectional and prospective correlations of overall suffering assessed at T1 with the outcomes assessed at T1 and T2, respectively (see Supplemental Table S5). Overall suffering was both cross-sectionally (*r*s = |0.09 to 0.57|) and prospectively (*r*s = |0.09 to 0.38|) correlated with worse health and well-being on each outcome (small to very large in magnitude); for most outcomes, the magnitude of its prospective correlation was smaller than its cross-sectional correlation. Across both sets of correlations, associations tended to be largest for indices of physical health, indices of mental health, and some indices of psychological well-being.

#### Primary analysis: associations of overall suffering with health and well-being outcomes

Consistent with Study 1, missing data on all study variables were imputed using multiple imputation by chained equations. Applying Rubin’s^43^ rule, we performed a series of linear regression analyses in which each of the 16 outcome variables at T2 was regressed on overall suffering assessed at T1 (one outcome at a time). Each model adjusted for all sociodemographic characteristics and the baseline value (or close proxy) of the respective outcome assessed at T1. All outcomes were continuous and standardized (*M* = 0, *SD* = 1). All tables that report the results of multivariable analyses include cutoffs for *p*-values both before and after Bonferroni correction. For reasons outlined in Study 1, we do not use Bonferroni correction as the primary lens for interpreting the results.

Results for the associations of overall suffering assessed at T1 with each of the subsequent health and well-being outcomes assessed at T2 are presented in Table 3. Overall suffering was associated with subsequently worse health and well-being on at least one outcome in each domain (small to large in magnitude), including all indices of physical health (βs = − 0.09 to 0.31, *p*s ≤ 0.001), sleepless days (β = 0.11, *p* < 0.001), both indices of mental health (βs = 0.11 to 0.13, *p*s ≤ 0.001), the psychological well-being indices of life satisfaction, happiness, and meaning in life (βs = − 0.09 to − 0.07, *p*s ≤ 0.017), the character strength of being oriented toward promoting good (β = − 0.09, *p* < 0.001), and satisfying relationships (β = − 0.08, *p* = 0.006). Sense of purpose (psychological well-being domain) and delay gratification (character strengths domain) were the only two outcomes for which overall suffering showed little evidence of association (*p*s > 0.05). Most results were comparable when the analyses were replicated using tertiles of overall suffering (with tertile 1 serving as the reference category), but associations for life satisfaction, happiness, meaning in life, and satisfying relationships attenuated to the null (see Supplemental Table S11). The complete-case analysis also yielded similar results (see Supplemental Table S7), except that overall suffering was no longer associated with happiness, meaning in life, and satisfying relationships, and evidence of a negative association emerged for delay gratification.

**Table 3:** Associations of overall suffering (T1) with health and well-being outcomes (T2) in Study 2 (flight attendant sample).

Outcome	Effect estimate		*E*-values	
	Reference	β [95% CI]	Effect estimate	CI limit
**Physical health**				
General health	0.00	− 0.09 [− 0.14, − 0.03]***	1.38	1.21
Physically unhealthy days	0.00	0.31 [0.25, 0.37]***	1.98	1.81
Pain-related limitations days	0.00	0.23 [0.17, 0.30]***	1.78	1.61
Disability days	0.00	0.27 [0.20, 0.34]***	1.88	1.70
Fatigue days (past 30 days)^#^	0.00	0.20 [0.14, 0.26]***	1.68	1.53
Vitality days	0.00	− 0.22 [− 0.28, − 0.16]***	1.75	1.59
**Health behavior**				
Sleepless days	0.00	0.11 [0.06, 0.16]***	1.44	1.28
**Mental health**				
Mentally unhealthy days	0.00	0.13 [0.07, 0.19]***	1.50	1.32
Depressed mood days^##^	0.00	0.11 [0.04, 0.17]***	1.43	1.24
**Psychological well-being**				
Life satisfaction	0.00	− 0.08 [− 0.14, − 0.02]*	1.37	1.17
Happiness	0.00	− 0.07 [− 0.12, − 0.01]*	1.32	1.12
Meaning in life	0.00	− 0.09 [− 0.15, − 0.04]***	1.40	1.22
Sense of purpose	0.00	− 0.04 [− 0.09, 0.01]	1.24	1.00
**Character strengths**				
Promote good	0.00	− 0.09 [− 0.15, − 0.04]***	1.40	1.24
Delay gratification	0.00	− 0.03 [− 0.08, 0.02]	1.20	1.00
**Social well-being**				
Satisfying relationships	0.00	− 0.08 [− 0.13, − 0.02]*	1.35	1.16

*β* standardized effect size, *CI* confidence interval. *n* = 1402 for all analyses. Multiple imputation was performed to impute missing data on the exposure, covariates, and outcomes. We ran a linear regression model to estimate a β for all outcomes (one outcome at a time). Each outcome was continuous and standardized (*M* = 0, *SD* = 1). All models adjusted for prior values of age, gender, sexual orientation, racial status, marital status, educational attainment, job tenure, child dependents, and older adult dependents assessed at T1. Unless otherwise indicated, all models also adjusted for the prior value of the respective outcome assessed at T1. If a prior value of an outcome was not available, we adjusted for the prior value of a variable that was most comparable to the outcome: ^#^fatigue days (past 7 days), ^##^depression symptoms (see Supplemental Table S10). *p < 0.05 before Bonferroni correction, ***p < 0.05 after Bonferroni correction (the *p*-value cutoff for Bonferroni correction was 0.05/16 = 0.003 for each outcome).

As with Study 1, we used *E*-values to assess the robustness of the results from the primary analysis to potential unmeasured confounding. *E*-values ranged from 1.20 to 1.98 for the effect estimates and 1.00 to 1.81 for the confidence interval limits closest to the null (see Table 3). For most outcomes, *E*-values suggested that some of the observed associations were at least modestly robust to residual confounding. The physical health domain generally had more compelling evidence for robustness than the other domains of health and well-being.

#### Secondary analysis: associations of suffering aspects with health and well-being outcomes

Similar to Study 1, we estimated the effects of the seven aspects of suffering assessed at T1 on the subsequent outcomes assessed at T2. Analytic models mirrored the primary analysis, except that in each model overall suffering was replaced with an aspect of suffering (modeled as continuous variables) as the exposure variable (one aspect of suffering and one outcome in each model). The results for this set of analyses are reported in Supplemental Table S12. The general pattern of results was largely comparable to those that emerged in the primary analysis. In most cases where overall suffering was associated with worse health and well-being on an outcome in the primary analysis (nine out of 14 outcomes), all seven aspects of suffering also showed evidence of association with the outcome (small to large in magnitude). Of the five outcomes that diverged from this trend, one aspect of suffering (i.e., length of suffering) was not associated with two of the outcomes (i.e., general health, satisfying relationships), two aspects of suffering (i.e., extent of suffering, length of suffering) were not associated with two of the outcomes (i.e., depressed mood days, happiness), and three aspects of suffering (i.e., extent of suffering, intensity of suffering, powerlessness over suffering) were not associated with one outcome (i.e., life satisfaction). Threats to personhood was associated with sense of purpose (small in magnitude), but there was little evidence to suggest that any aspect of suffering was associated with the character strength of delay gratification.

### Summary

In this study of flight attendants, we found modest to strong evidence indicating that overall suffering was associated with worse health and well-being across multiple domains of functioning approximately 2 years later. Effect sizes were generally larger for indices of physical health, with somewhat smaller effects emerging for indices on other health and well-being domains. Although the FAHS dataset did not contain information about sources or objects of suffering, this pattern of findings points to the possibility that many individuals may have responded to the suffering items by reflecting principally on their physical health.

Findings were largely similar for the aspects of suffering, with some variation in the consistency of associations across outcomes. For example, some aspects of suffering (e.g., length of suffering) were not associated with certain outcomes that were predicted by overall suffering (e.g., general health, happiness), and one aspect of suffering (e.g., threats to personhood) was associated with an outcome (e.g., sense of purpose) that did not show any evidence of association with overall suffering. Taken together, these findings provide largely consistent evidence suggesting that suffering is associated with worse health and well-being.

## General discussion

Employers and occupational health psychologists are increasingly recognizing the relevance of suffering in the workplace^57,58^. Since suffering denotes a state of worsened subjective well-being, it is likely to introduce risk to employee and employer alike. To date, however, much of the research on suffering and individual well-being has been conducted among Western samples of older adults with physical illnesses. Expanding the existing knowledge base in this area, we used longitudinal data from samples of garment factory workers (Study 1) and flight attendants (Study 2) to examine the associations of suffering with a diverse array of health and well-being outcomes among working adults.

The findings of the present research suggest that the implications of suffering for health and well-being may vary by population (amongst other factors). Whereas overall suffering showed evidence of association with few (2/16) outcomes in the garment factory worker sample (Study 1), it was associated with almost all (14/16) outcomes in the flight attendant worker sample (Study 2). This difference in the pattern of results across the two studies could be due to various methodological factors, such as differences in sample characteristics, measures used to assess outcomes, or timing of assessments. For example, the average age of participants in Study 1 (mean approximating young adulthood) was nearly half that of the participants in Study 2 (mean approximating middle adulthood), with our findings showing some support for the idea that suffering is often a ‘problem’ of aging^59^. This could be one of the reasons why much of the existing literature on suffering and health and well-being outcomes has focused on older adults.

Importantly, the findings of Study 2 are among the first to show that the potential implications of suffering for health and well-being extend beyond older adults dealing with physical health problems or illness (e.g., terminal cancer), demonstrating that suffering also has the capacity to degrade different facets of health and well-being among middle-aged adults who are healthy enough to work. Although our findings suggest that suffering may have more wide-ranging implications for health and well-being at older ages, Study 1 provided further evidence indicating that the negative effects of suffering are not limited to older populations. This is consistent with recent studies involving younger adults^13^, highlighting the importance of research exploring suffering across the life course.

Our findings provide useful empirical evidence for further addressing the common notion that suffering can lead to growth in character. Contrary to theory^19^, qualitative literature^60^, and some cross-sectional evidence^30^, both Studies 1 and 2 provided little evidence to suggest that overall suffering was associated with subsequent increases in characterological orientation to promote the good or ability to delay gratification. However, these findings align with previous longitudinal research in which overall suffering did not predict short-term improvements in character strengths^13^, and also resonate more broadly with research that has documented inconclusive evidence for character growth in the wake of stressful life events^61^. One potential explanation for these findings is that character growth in response to suffering may require more time to develop, given that the lag between the assessment of suffering and the outcomes in Studies 1 and 2 was approximately 2 years. There may also be considerable heterogeneity in growth with suffering, such that the majority of people may not experience such growth, but some might. If this is so, understanding the conditions that facilitate character growth would be important.

We found that suffering was associated with a subsequent decrease in orientation to promote the good in Study 2, but this was not the case in Study 1. The finding in Study 2 may have been a function of the unique pandemic-related circumstances that people were experiencing around the time the outcomes were assessed. For example, the public health measures that were implemented during the COVID-19 pandemic (e.g., stay-at-home orders, social distancing mandates) may have limited flight attendants’ opportunities to engage in altruistic behaviors. An alternative possibility is that the challenges precipitated by the public health crisis might have prompted flight attendants who reported higher levels of overall suffering at baseline to turn ‘inward’ to conserve psychological resources that were threatened (e.g., feelings of control)^12^. Research is needed to test such theorizing empirically, as well as to examine relevant moderators that might contribute to transforming experiences of suffering into the development of character strengths.

The results from the secondary analyses involving each aspect of suffering provided insight into the complex linkages between suffering and facets of health and well-being. In Studies 1 and 2, some outcomes for which there was evidence of an association with overall suffering were not predicted by one or more aspects of suffering. For example, in Study 1 overall suffering was associated with an increase in subsequent depressed mood, but only two aspects of suffering (i.e., powerlessness over suffering, pervasiveness of suffering) were associated with depressed mood. There were also cases in both studies where one or more aspects of suffering showed evidence of association with an outcome when overall suffering did not. For instance, in Study 1 a null association was found between overall suffering and general health, but length of suffering and threats to personhood were associated with a subsequent decrease in general health. Interestingly, we also found that threats to personhood were associated with a subsequent increase in satisfying relationships in Study 1, even though other aspects of suffering and overall suffering were unrelated to satisfying relationships. Unlike other aspects of suffering (e.g., powerlessness, intensity), perceived threats to personhood may be unique in that they might motivate adaptive interpersonal approach-oriented responses (i.e., seeking social support) to attenuate such experiences^62^. Overall, our findings correspond with previous research that has found the associations between some aspects of suffering and well-being outcomes often differ from those found for overall suffering^2,13^. Based on evidence accumulated so far, a more fine-grained understanding of the relations between suffering and well-being might be achieved by focusing on different aspects of suffering individually.

The pattern of associations for aspects of suffering varied across the two studies, indicating that some aspects of suffering may have a more prominent role in shaping health and well-being outcomes in certain populations compared to others. There may be several reasons for the variation that we observed across the two samples, such as differences in sociodemographics (e.g., age), cultural understandings of suffering, and approaches to dealing with suffering. One potentially important cultural difference could consist in the religious affiliations prevalent in the regions we examined. Most Sri Lankans adhere to Buddhism^63^, which tends to treat suffering differently than the Christian approaches that have been historically dominant in Anglo-American contexts^64^. Moreover, the concept of suffering and the measure that we used to assess suffering in our studies was derived principally from Western scholarship, which may be less sensitive to Buddhist (and other Eastern) perspectives about suffering. For example, our understanding of suffering as an undesired experience of a lost good may contrast with the Buddhist notion that suffering arises from our cravings for something impermanent^65^. Contrasts like these could have impacted how participants responded to one or more of the suffering items that were used in the present research.

We also found that some aspects of suffering were more consistently associated with worse health and well-being outcomes across both studies, including threats to personhood (2/16 outcomes in Study 1, 15/16 outcomes in Study 2), pervasiveness of suffering (2/16 outcomes in Study 1, 14/16 outcomes in Study 2), and powerlessness over suffering (2/16 outcomes in Study 1, 13/16 outcomes in Study 2). This trend aligns with earlier studies that have reported these aspects of suffering as among the most consistent predictors of various health and well-being outcomes^2,13^. Taken together, these findings suggest that some aspects of suffering may have stronger and more wide-ranging consequences for health and well-being compared to others.

### Limitations and future research

There are several limitations associated with the present set of studies. First, participants in each study were conveniently sampled, and it is unclear whether the findings reported herein can be generalized to the populations from which the samples were drawn. Further study is needed to determine whether our findings replicate in more representative samples and working adults employed in other professions (e.g., healthcare workers).

Second, the rate of attrition in each study was high, which can result in selection bias if participants in the analytic sample differ systematically from those who did not participate in both the T1 and T2 surveys. Descriptive analyses in each study indicated that there were some differences between the baseline characteristics of those who participated in both waves and those who did not (see Supplemental Tables S1 and S9), introducing the possibility that measured or unmeasured factors may have biased the results and weakened the generalizability of our findings.

Third, the WWBS and FAHS are designed to assess a wide range of domains relevant to health and well-being. This approach enabled us to evaluate many different indicators of health and well-being in each sample, but outcomes in both studies were limited to single-item measures. Although the items that we used for the outcomes were taken from measures that have been well-validated and widely implemented in research, follow-up studies are needed with measures that more comprehensively cover the conceptual breadth of various outcomes that we examined. Along similar lines, the WWBS and FAHS did not obtain information about sources or objects of suffering, which could be important for understanding experiences of suffering, downstream consequences of suffering, and mechanisms that might influence health and well-being outcomes. Hence, our findings might be enriched by research that identifies and distinguishes sources and objects of suffering.

Fourth, our findings are based exclusively on self-report responses, which could be impacted by different forms of response bias (e.g., social desirability). Although this set of studies represents an important step toward building a richer body of knowledge about suffering and how it might affect a wide range of *subjectively* reported facets of health and well-being, future research would do well to integrate data derived from multiple sources (e.g., healthcare professionals) and methods (e.g., clinical assessments, psychophysiology) to provide a complementary and more balanced set of metrics that can be used to evaluate the effects of suffering on individual health and well-being.

Fifth, the T2 outcomes in Study 2 were assessed in the early part of the COVID-19 pandemic, when people in many parts of the world were adapting to drastic changes in daily life and experiencing a wide range of unprecedented challenges^66,67^. Although we are unable to determine the extent to which the COVID-19 pandemic itself affected responses to the T2 outcomes in Study 2, those findings should be interpreted with some caution because of the general (e.g., social distancing) and population-specific (e.g., additional job demands imposed on flight crew) impacts that the public health crisis might have had on flight attendants in the analytic sample. Given the largely unique findings that emerged in Studies 1 and 2, research which is sensitive to workplace context may be important for understanding the implications of suffering for the health and well-being of worker populations employed within different industries. Special attention should be dedicated to healthcare workers, particularly those who engage frequently with people who are suffering (e.g., terminal patients), given that experiences of suffering among workers in the healthcare industry could negatively affect the quality of care that their patients receive^68^.

## Conclusion

The studies reported herein are among the first to estimate the effects of suffering on different facets of health and well-being among working adults. The findings contribute to a growing body of empirical literature suggesting that the implications of suffering for health and well-being extend beyond clinical populations of adults who are dealing with chronic physical health issues. Although further research is needed to accumulate additional evidence about the consequences of suffering for employee well-being, their performance at work, and organizational functioning overall, our findings could be useful to various organizational stakeholders (e.g., management, occupational health psychologists) who are involved in planning and implementing strategies to support worker well-being.

## Supplementary Information

Supplementary Information.

## Data Availability

The datasets used and/or analyzed are available from the corresponding author on reasonable request.
