# Supplementary material for: Associations of suffering with facets of health and well-being among working adults: longitudinal evidence from two samples
Source: Sci Rep. 2022 Nov 22;12:20141. doi: 10.1038/s41598-022-24497-8 (PMC9684157; doi:10.1038/s41598-022-24497-8)
Supplement: Supplementary file 1 — Supplementary Information. [file 41598_2022_24497_MOESM1_ESM.docx]

Associations of Suffering with Facets of Health and Well-Being Among Working Adults: Longitudinal Evidence from Two Samples

**SUPPLEMENTAL MATERIAL**

| Supplemental Table S1  *Baseline Characteristics of Participants in Study 1 (Factory Worker Sample) Who Completed Both T1 and T2 Surveys and Those Who Did Not* | | | | | | | | | | | |
| --- | --- | --- | --- | --- | --- | --- | --- | --- | --- | --- | --- |
| Characteristic | Total (*n* = 1,258) | | |  | T1 and T2 (*n* = 344) | |  | T1 only (*n* = 914) | | *p*-value | *d* |
|  | *n* | % | *M* ± *SD* (range) |  | % | *M* ± *SD* (range) |  | % | *M* ± *SD* (range) |  |  |
| **Sociodemographics** |  |  |  |  |  |  |  |  |  |  |  |
| Age (years) | 1,249 |  | 30.61 ± 9.16 (18–70) |  |  | 31.71 ± 9.04 (18–55) |  |  | 30.19 ± 9.18 (18–70) | .008 | .17 |
| Gender | 1,255 |  |  |  |  |  |  |  |  | < .001 | .19 |
| Female | 724 | 57.69 |  |  | 65.41 |  |  | 54.77 |  |  |  |
| Male | 531 | 42.31 |  |  | 34.59 |  |  | 45.23 |  |  |  |
| Ethnic status | 1,216 |  |  |  |  |  |  |  |  | .586 | .03 |
| Sinhalese | 1,095 | 90.05 |  |  | 90.96 |  |  | 89.71 |  |  |  |
| Other | 121 | 9.95 |  |  | 9.04 |  |  | 10.29 |  |  |  |
| Marital status | 1,251 |  |  |  |  |  |  |  |  | .418 | .05 |
| Married | 728 | 58.19 |  |  | 60.17 |  |  | 57.44 |  |  |  |
| Other | 523 | 41.81 |  |  | 39.83 |  |  | 42.56 |  |  |  |
| Educational attainment | 1,201 |  |  |  |  |  |  |  |  | .224 | .07 |
| Up to high school equivalency | 488 | 40.63 |  |  | 43.60 |  |  | 39.52 |  |  |  |
| Some postsecondary education or higher | 713 | 59.37 |  |  | 56.40 |  |  | 60.48 |  |  |  |
| Child dependents | 1,147 |  |  |  |  |  |  |  |  | .095 | .10 |
| No | 616 | 53.71 |  |  | 49.52 |  |  | 55.26 |  |  |  |
| Yes | 531 | 46.29 |  |  | 50.48 |  |  | 44.74 |  |  |  |
| Older adult dependents | 1,161 |  |  |  |  |  |  |  |  | .549 | .04 |
| No | 577 | 49.70 |  |  | 48.10 |  |  | 50.30 |  |  |  |
| Yes | 584 | 50.30 |  |  | 51.90 |  |  | 49.70 |  |  |  |
| Employment tenure | 1,258 |  |  |  |  |  |  |  |  | .049 | .11 |
| ≤ 5 years | 940 | 74.72 |  |  | 70.64 |  |  | 76.26 |  |  |  |
| > 5 years | 318 | 25.28 |  |  | 29.36 |  |  | 23.74 |  |  |  |
| **Suffering** |  |  |  |  |  |  |  |  |  |  |  |
| Overall suffering | 1,000 |  | 3.67 ± 2.77 (0–10) |  |  | 3.96 ± 2.86 (0–10) |  |  | 3.57 ± 2.74 (0–10) | .052 | .14 |
| **Physical health** |  |  |  |  |  |  |  |  |  |  |  |
| General health | 1,226 |  | 3.39 ± 1.18 (1–5) |  |  | 3.34 ± 1.22 (1–5) |  |  | 3.41 ± 1.16 (1–5) | .356 | .06 |
| Physical health | 1,207 |  | 7.56 ± 2.77 (0–10) |  |  | 7.57 ± 2.74 (0–10) |  |  | 7.56 ± 2.78 (0–10) | .934 | .00 |
| Pain-related limitations days | 1,166 |  | 1.56 ± 4.05 (0–30) |  |  | 1.08 ± 3.10 (0–30) |  |  | 1.73 ± 4.33 (0–30) | .005 | .16 |
| Disability days | 1,149 |  | 2.21 ± 4.92 (0–30) |  |  | 2.01 ± 4.50 (0–30) |  |  | 2.29 ± 5.06 (0–30) | .361 | .06 |
| **Health behavior** |  |  |  |  |  |  |  |  |  |  |  |
| Sleepless days | 1,179 |  | 3.17 ± 5.52 (0–30) |  |  | 2.51 ± 4.79 (0–30) |  |  | 3.43 ± 5.75 (0–30) | .006 | .17 |
| Current smoking | 1,227 |  |  |  |  |  |  |  |  | .003 | .17 |
| No | 1,102 | 89.81 |  |  | 94.08 |  |  | 88.19 |  |  |  |
| Yes | 125 | 10.19 |  |  | 5.92 |  |  | 11.81 |  |  |  |
| **Mental health** |  |  |  |  |  |  |  |  |  |  |  |
| Mental health | 1,186 |  | 7.77 ± 2.71 (0–10) |  |  | 7.68 ± 2.80 (0–10) |  |  | 7.80 ± 2.67 (0–10) | .509 | .04 |
| Depressed mood days | 1,161 |  | 3.16 ± 5.96 (0–30) |  |  | 2.79 ± 5.70 (0–30) |  |  | 3.30 ± 6.05 (0–30) | .179 | .09 |
| **Psychological well-being** |  |  |  |  |  |  |  |  |  |  |  |
| Life satisfaction | 1,122 |  | 6.30 ± 3.56 (0–10) |  |  | 6.56 ± 3.55 (0–10) |  |  | 6.21 ± 3.56 (0–10) | .135 | .10 |
| Happiness | 1,201 |  | 6.83 ± 3.51 (0–10) |  |  | 6.80 ± 3.61 (0–10) |  |  | 6.84 ± 3.48 (0–10) | .845 | .01 |
| Meaning in life | 1,109 |  | 8.05 ± 2.78 (0–10) |  |  | 8.12 ± 2.77 (0–10) |  |  | 8.02 ± 2.78 (0–10) | .599 | .04 |
| Sense of purpose | 1,197 |  | 8.42 ± 2.58 (0–10) |  |  | 8.40 ± 2.65 (0–10) |  |  | 8.43 ± 2.55 (0–10) | .836 | .01 |
| **Character strengths** |  |  |  |  |  |  |  |  |  |  |  |
| Promote good | 1,160 |  | 8.21 ± 2.81 (0–10) |  |  | 8.27 ± 2.68 (0–10) |  |  | 8.18 ± 2.86 (0–10) | .638 | .03 |
| Delay gratification | 1,184 |  | 8.28 ± 2.71 (0–10) |  |  | 8.26 ± 2.69 (0–10) |  |  | 8.28 ± 2.73 (0–10) | .868 | .01 |
| **Social well-being** |  |  |  |  |  |  |  |  |  |  |  |
| Satisfying relationships | 1,184 |  | 7.58 ± 3.11 (0–10) |  |  | 7.52 ± 3.13 (0–10) |  |  | 7.60 ± 3.11 (0–10) | .699 | .03 |
| Content relationships | 1,175 |  | 7.84 ± 3.00 (0–10) |  |  | 7.67 ± 3.18 (0–10) |  |  | 7.91 ± 2.92 (0–10) | .231 | .08 |
| *Note*. *d* = Cohen’s *d*, *M* = mean, *SD* = standard deviation. Percentages refer to the proportion of individuals within each inclusion category with that characteristic. *p*-values come from independent samples *t*-tests or *χ*^2^ tests that were used to examine the mean levels of the characteristic or the proportion of individuals within the retained and lost to follow-up categories with that characteristic. | | | | | | | | | | | |

| Supplemental Table S2  *List of Items Used for Exposure and Outcome Variables in Study 1 (Factory Worker Sample)* | | | |
| --- | --- | --- | --- |
| Variable | Item/question | Response scale | Assessment |
| **Suffering**^1^ |  |  | T1 |
| Extent of suffering | To what extent are you suffering? | 0 (*Not suffering at all*) to 10 (*Suffering terribly*) |  |
| Intensity of suffering | The intensity of my suffering feels intolerable. | 0 (*Do not agree*) to 10 (*Completely agree*) |  |
| Length of suffering | The length of time I have been suffering or expect to suffer, feels intolerable. | 0 (*Do not agree*) to 10 (*Completely agree*) |  |
| Powerlessness over suffering | I feel powerless to stop my current suffering. | 0 (*Do not agree*) to 10 (*Completely agree*) |  |
| Pervasiveness of suffering | The suffering I have been experiencing affects all aspects of my life. | 0 (*Do not agree*) to 10 (*Completely agree*) |  |
| Disruption to purposes | Certain purposes of my life have been badly disrupted because of my suffering. | 0 (*Do not agree*) to 10 (*Completely agree*) |  |
| Threats to personhood | My experience of suffering threatens who I am as a person. | 0 (*Do not agree*) to 10 (*Completely agree*) |  |
| **Physical health** |  |  |  |
| General health^2,3^ | Would you say that in general your health is… | 1 (*Poor*) to 5 (*Excellent*) | T1 & T2 |
| Physical health^4^ | In general, how would you rate your physical health? | 0 (*Poor*) to 10 (*Excellent*) | T1 & T2 |
| Pain-related limitations days^2,3^ | During the past 30 days, for about how many days did pain make it hard for you to do your usual activities, such as self-care, work, or recreation? | 0 to 30 (*Days*) | T1 |
| Pain-related limitations^5^ | Does pain make it hard for you to do your usual activities? | 0 (*Never*) to 10 (*Always*) | T2 |
| Disability days^2,3^ | During the past 30 days, for about how many days did poor physical or mental health keep you from doing your usual activities, such as self-care, work, or recreation? | 0 to 30 (*Days*) | T1 & T2 |
| **Health behavior** |  |  |  |
| Current smoking | Do you currently smoke tobacco products? | No vs. Yes | T1 & T2 |
| Sleepless days^2,3^ | During the past 30 days, how many days have you not gotten enough rest or sleep? | 0 to 30 (*Days*) | T1 |
| Adequate sleep | Thinking about the last 2 weeks, how many hours did you usually sleep on a work night? | No (< 7 hours) vs. Yes (≥ 7 hours) | T2 |
| **Mental health** |  |  |  |
| Mental health^4^ | In general, how would you rate your mental health? | 0 (*Poor*) to 10 (*Excellent*) | T1 & T2 |
| Depressed mood days^2,3^ | During the past 30 days, for about how many days did you feel sad or depressed? | 0 to 30 (*Days*) | T1 |
| Depressed mood^5^ | Are you depressed? (Depression is a consistent change in mood lasting for at least two weeks, and includes feeling sad, tired, having low energy, trouble sleeping, difficulty making decisions, and/or feeling worthless.) | 0 (*Not at all depressed*) to 10 (*Very depressed*) | T2 |
| **Psychological well-being** |  |  |  |
| Life satisfaction^4^ | Overall, how satisfied are you with life as a whole these days? | 0 (*Not at all satisfied*) to 10 (*Completely satisfied*) | T1 & T2 |
| Happiness^4^ | In general, how happy or unhappy do you usually feel? | 0 (*Extremely unhappy*) to 10 (*Extremely happy*) | T1 & T2 |
| Meaning in life^4^ | Overall, to what extent do you feel the things you do in your life are worthwhile? | 0 (*Not at all worthwhile*) to 10 (*Completely worthwhile*) | T1 & T2 |
| Sense of purpose^4^ | I understand my purpose in life. | 0 (*Strongly disagree*) 10 (*Strongly agree*) | T1 & T2 |
| **Character strengths** |  |  |  |
| Promote good^4^ | I always act to promote good in all circumstances, even in difficult and challenging situations. | 0 (*Strongly disagree*) 10 (*Strongly agree*) | T1 & T2 |
| Delay gratification^4^ | I am always able to give up some happiness now for greater happiness later. | 0 (*Strongly disagree*) 10 (*Strongly agree*) | T1 & T2 |
| **Social well-being** |  |  |  |
| Satisfying relationships^4^ | My relationships are as satisfying as I would want them to be. | 0 (*Strongly disagree*) 10 (*Strongly agree*) | T1 & T2 |
| Content relationships^4^ | I am content with my friendships and relationships. | 0 (*Strongly disagree*) 10 (*Strongly agree*) | T1 & T2 |
| *Note*. Personal Suffering Assessment^1^; Centers for Disease Control and Prevention Health-Related Quality of Life 14^2,3^; Flourishing Index^4^; Adapted from Well-Being Assessment^5^. | | | |

| Supplemental Table S3  *Distribution of Participant Characteristics in the Analytic Samples for Study 1 (Factory Worker Sample) and Study 2 (Flight Attendant Sample)* | | | | | |
| --- | --- | --- | --- | --- | --- |
| Variable | Study 1: Factory worker sample  (*n* = 344) | |  | Study 2: Flight attendant sample  (*n* = 1,402) | |
|  | *n* (%) | *M* ± *SD* (range) |  | *n* (%) | *M* ± *SD* (range) |
| **T1** |  |  |  |  |  |
| ***Sociodemographics*** |  |  |  |  |  |
| Age (years) | 344 | 31.71 ± 9.04 (18–55) |  | 1,398 | 56.09 ± 6.10 (46–75) |
| Gender | 344 |  |  | 1,402 |  |
| Female | 225 (65.41) |  |  | 1,155 (82.38) |  |
| Male | 119 (34.59) |  |  | 247 (17.62) |  |
| Sexual orientation | ~~-~~ |  |  | 1,398 |  |
| Heterosexual | - |  |  | 1,238 (88.56) |  |
| Other | - |  |  | 160 (11.44) |  |
| Racial status | - |  |  | 1,399 |  |
| White | - |  |  | 1,181 (84.42) |  |
| Other | - |  |  | 218 (15.58) |  |
| Ethnic status | 332 |  |  | - |  |
| Sinhalese | 302 (90.96) |  |  | - |  |
| Other | 30 (9.04) |  |  | - |  |
| Marital status | 344 |  |  | 1,238 |  |
| Married | 207 (60.17) |  |  | 654 (52.83) |  |
| Other | 137 (39.83) |  |  | 584 (47.17) |  |
| Educational attainment | 328 |  |  | 1,239 |  |
| Up to high school equivalency | 143 (43.60) |  |  | - |  |
| Some postsecondary education or higher | 185 (56.40) |  |  | - |  |
| Up to completion of undergraduate degree | - |  |  | 1,094 (88.30) |  |
| Graduate school/advanced degree | - |  |  | 145 (11.70) |  |
| Child dependents | 311 |  |  | 1,205 |  |
| No | 154 (49.52) |  |  | 991 (82.24) |  |
| Yes | 157 (50.48) |  |  | 214 (17.76) |  |
| Older adult dependents | 316 |  |  | 1,234 |  |
| No | 152 (48.10) |  |  | 1,053 (85.33) |  |
| Yes | 164 (51.90) |  |  | 181 (14.67) |  |
| Employment tenure | 344 |  |  | 1,396 |  |
| ≤ 5 years | 243 (70.64) |  |  | 1,337 (95.77) |  |
| > 5 years | 101 (29.36) |  |  | 59 (4.23) |  |
| ***Suffering*** |  |  |  |  |  |
| Overall suffering (α_Study 1_ = .88; α_Study 2_ = .95) | 264 | 3.96 ± 2.86 (0–10) |  | 1,334 | 2.21 ± 2.41 (0–9.71) |
| Extent of suffering | 326 | 3.01 ± 3.10 (0–10) |  | 1,361 | 2.84 ± 2.56 (0–10) |
| Intensity of suffering | 306 | 4.34 ± 3.81 (0–10) |  | 1,358 | 1.86 ± 2.35 (0–10) |
| Length of suffering | 298 | 4.16 ± 3.76 (0–10) |  | 1,351 | 1.91 ± 2.50 (0–10) |
| Powerlessness over suffering | 312 | 4.24 ± 3.95 (0–10) |  | 1,353 | 1.98 ± 2.73 (0–10) |
| Pervasiveness of suffering | 308 | 4.41 ± 4.01 (0–10) |  | 1,352 | 2.67 ± 3.19 (0–10) |
| Disruption to purposes | 315 | 4.37 ± 3.80 (0–10) |  | 1,350 | 2.46 ± 3.02 (0–10) |
| Threats to personhood | 308 | 3.79 ± 3.72 (0–10) |  | 1,349 | 1.80 ± 2.70 (0–10) |
| ***Physical health*** |  |  |  |  |  |
| General health | 331 | 3.34 ± 1.22 (1–5) |  | 1,383 | 3.67 ± 0.92 (1–5) |
| Physical health | 331 | 7.57 ± 2.74 (0–10) |  | - | - |
| Physically unhealthy days | - | - |  | 1,366 | 5.12 ± 7.90 (0–30) |
| Pain-related limitations days | 314 | 1.08 ± 3.10 (0–30) |  | 1,351 | 5.88 ± 8.87 (0–30) |
| Disability days | 312 | 2.01 ± 4.50 (0–30) |  | 1,342 | 4.08 ± 7.33 (0–30) |
| Fatigue days (past 7 days) | - | - |  | 1,305 | 2.48 ± 1.35 (1–5) |
| Vitality days | - | - |  | 1,186 | 12.53 ± 9.52 (0–30) |
| ***Health behavior*** |  |  |  |  |  |
| Sleepless days | 324 | 2.51 ± 4.79 (0–30) |  | 1,193 | 11.61 ± 8.82 (0–30) |
| Current smoking | 338 |  |  | - | - |
| No | 318 (94.08) |  |  |  |  |
| Yes | 20 (5.92) |  |  |  |  |
| ***Mental health*** |  |  |  |  |  |
| Mentally unhealthy days | - | - |  | 1,354 | 5.13 ± 7.49 (0–30) |
| Depression symptoms (α = .86) | - | - |  | 1,270 | 5.79 ± 4.80 (0–26) |
| Mental health | 324 | 7.68 ± 2.80 (0–10) |  | - | - |
| Depressed mood days | 313 | 2.79 ± 5.70 (0–30) |  | - | - |
| ***Psychological well-being*** |  |  |  |  |  |
| Life satisfaction | 303 | 6.56 ± 3.55 (0–10) |  | 1,338 | 7.26 ± 1.97 (0–10) |
| Happiness | 329 | 6.80 ± 3.61 (0–10) |  | 1,337 | 7.31 ± 1.75 (0–10) |
| Meaning in life | 294 | 8.12 ± 2.77 (0–10) |  | 1,342 | 7.69 ± 1.90 (0–10) |
| Sense of purpose | 325 | 8.40 ± 2.65 (0–10) |  | 1,337 | 7.23 ± 2.26 (0–10) |
| ***Character strengths*** |  |  |  |  |  |
| Promote good | 311 | 8.27 ± 2.68 (0–10) |  | 1,336 | 8.05 ± 1.53 (0–10) |
| Delay gratification | 317 | 8.26 ± 2.69 (0–10) |  | 1,325 | 7.38 ± 1.85 (0–10) |
| ***Social well-being*** |  |  |  |  |  |
| Satisfying relationships | 318 | 7.52 ± 3.13 (0–10) |  | 1,329 | 6.50 ± 2.51 (0–10) |
| Content relationships | 323 | 7.67 ± 3.18 (0–10) |  | - | - |
| **T2** |  |  |  |  |  |
| ***Physical health*** |  |  |  |  |  |
| General health | 332 | 3.29 ± 1.10 (1–5) |  | 1,313 | 3.51 ± 0.90 (1–5) |
| Physical health | 340 | 7.77 ± 2.27 (0–10) |  | - | - |
| Physically unhealthy days | - | - |  | 1,311 | 5.45 ± 8.37 (0–30) |
| Pain-related limitations days | - | - |  | 1,312 | 4.86 ± 8.16 (0–30) |
| Pain-related limitations | 336 | 3.06 ± 3.12 (0–10) |  | - | - |
| Disability days | 292 | 2.15 ± 4.90 (0–30) |  | 1,313 | 4.89 ± 7.78 (0–30) |
| Fatigue days (past 30 days) | - | - |  | 1,293 | 2.88 ± 1.14 (1–5) |
| Vitality days | - | - |  | 1,263 | 14.95 ± 10.28 (0–30) |
| ***Health behavior*** |  |  |  |  |  |
| Sleepless days | - | - |  | 1,314 | 9.54 ± 8.89 (0–30) |
| Adequate sleep | 315 |  |  | - |  |
| No (< 7 hours) | 146 (46.35) |  |  | - |  |
| Yes (≥ 7 hours) | 169 (53.65) |  |  | - |  |
| Current smoking | 334 |  |  | - |  |
| No | 312 (93.41) |  |  | - |  |
| Yes | 22 (6.59) |  |  | - |  |
| ***Mental health*** |  |  |  | - |  |
| Mentally unhealthy days | - | - |  | 1,313 | 7.05 ± 8.47 (0–30) |
| Depressed mood days | - | - |  | 1,313 | 6.23 ± 8.31 (0–30) |
| Mental health | 335 | 7.69 ± 2.57 (0–10) |  | - | - |
| Depressed mood | 337 | 3.23 ± 3.09 (0–10) |  | - | - |
| ***Psychological well-being*** |  |  |  |  |  |
| Life satisfaction | 341 | 7.30 ± 2.73 (0–10) |  | 1,367 | 6.40 ± 2.20 (0–10) |
| Happiness | 333 | 7.02 ± 2.54 (0–10) |  | 1,355 | 7.19 ± 1.82 (0–10) |
| Meaning in life | 331 | 8.39 ± 2.20 (0–10) |  | 1,353 | 7.56 ± 1.90 (0–10) |
| Sense of purpose | 341 | 8.82 ± 2.02 (0–10) |  | 1,346 | 7.30 ± 2.11 (0–10) |
| ***Character strengths*** |  |  |  |  |  |
| Promote good | 337 | 8.46 ± 2.24 (0–10) |  | 1,338 | 8.22 ± 1.45 (0–10) |
| Delay gratification | 338 | 8.72 ± 2.13 (0–10) |  | 1,337 | 7.64 ± 1.74 (0–10) |
| ***Social well-being*** |  |  |  |  |  |
| Satisfying relationships | 339 | 7.10 ± 2.81 (0–10) |  | 1,331 | 6.99 ± 2.34 (0–10) |
| Content relationships | 337 | 6.74 ± 2.68 (0–10) |  | - | - |
| *Note*. *M* = mean, *SD* = standard deviation, α = alpha (estimated internal consistency reliability). | | | | | |

| Supplemental Table S4  *Correlations Between Aspects of Suffering in Study 1 (Factory Worker Sample^#^) and Study 2 (Flight Attendant Sample^##^)* | | | | | | | |
| --- | --- | --- | --- | --- | --- | --- | --- |
| Aspect of suffering | (1) | (2) | (3) | (4) | (5) | (6) | (7) |
| (1) Extent of suffering |  | .38 [.28, .47]* | .26 [.15, .36]* | .32 [.21, .42]* | .34 [.24, .44]* | .39 [.29, .48]* | .27 [.16, .37]* |
| (2) Intensity of suffering | .75 [.73, .78]* |  | .81 [.76, .84]* | .61 [.53, .67]* | .54 [.45, .61]* | .49 [.39, .57]* | .45 [.35, .54]* |
| (3) Length of suffering | .76 [.74, .78]* | .81 [.79, .82]* |  | .62 [.55, .69]* | .58 [.50, .66]* | .54 [.46, .62]* | .53 [.44, .61]* |
| (4) Powerlessness over suffering | .66 [.63, .69]* | .66 [.62, .69]* | .76 [.73, .78]* |  | .64 [.56, .70]* | .54 [.45, .61]* | .63 [.56, .70]* |
| (5) Pervasiveness of suffering | .76 [.74, .79]* | .71 [.68, .73]* | .74 [.71, .76]* | .77 [.75, .79]* |  | .66 [.59, .72]* | .55 [.47, .63]* |
| (6) Disruption to purposes | .75 [.73, .77]* | .70 [.67, .73]* | .75 [.73, .77]* | .76 [.74, .78]* | .87 [.85, .88]* |  | .62 [.54, .68]* |
| (7) Threats to personhood | .68 [.65, .71]* | .69 [.66, .72]* | .74 [.72, .77]* | .74 [.72, .77]* | .78 [.76, .80]* | .83 [.81, .84]* |  |
| *Note*. **p* < .001. Correlations above the diagonal correspond with Study 1 (factory worker sample), and correlations below the diagonal correspond with Study 2 (flight attendant sample). 95% confidence intervals presented in brackets. ^#^*n* = 283–326 for analyses, ^##^*n* = 1,342–1,361 for analyses. | | | | | | | |

| Supplemental Table S5  *Cross-sectional and Prospective Pearson Correlations of Overall Suffering with Outcomes in Study 1 (Factory Worker Sample) and Study 2 (Flight Attendant Sample)* | | |
| --- | --- | --- |
| Outcome (T1) | Overall suffering (T1) | |
|  | Study 1: Factory worker sample^#^ | Study 2: Flight attendant sample^##^ |
| ***Physical health*** |  |  |
| General health | -.24 [-.35, -.13]*** | -.49 [-.53, -.44]*** |
| Physical health | -.03 [-.15, .09] | - |
| Physically unhealthy days | - | .49 [.45, .53]*** |
| Pain-related limitations days | .15 [.03, .27]* | .54 [.50, .57]*** |
| Disability days | .29 [.18, .40]*** | .57 [.53, .60]*** |
| Fatigue days (past 7 days) | - | .48 [.43, .52]*** |
| Vitality days | - | -.44 [-.48, -.39]*** |
| ***Health behavior*** |  |  |
| Sleepless days | .10 [-.02, .22] | .22 [.16, .27]*** |
| Current smoking (No = 0, Yes = 1) | .02 [-.10, .14] | - |
| ***Mental health*** |  |  |
| Mentally unhealthy days | - | .52 [.48, .56]*** |
| Depression symptoms | - | .56 [.52, .59]*** |
| Mental health | -.15 [-.27, -.03]* | - |
| Depressed mood days | .24 [.12, .35]*** | - |
| ***Psychological well-being*** |  |  |
| Life satisfaction | -.11 [-.23, .01] | -.52 [-.56, -.48]*** |
| Happiness | -.12 [-.24, .00] | -.46 [-.50, -.41]*** |
| Meaning in life | -.19 [-.31, -.07]** | -.38 [-.42, -.33]*** |
| Sense of purpose | -.16 [-.28, -.04]** | -.28 [-.33, -.23]*** |
| ***Character strengths*** |  |  |
| Promote good | -.17 [-.29, -.05]** | -.09 [-.14, -.04]** |
| Delay gratification | -.10 [-.22, .02] | -.10 [-.16, -.05]*** |
| ***Social well-being*** |  |  |
| Satisfying relationships | -.06 [-.18, .07] | -.32 [-.37, -.27]*** |
| Content relationships | -.07 [-.19, .05] | - |
| Outcome (T2) | Overall suffering (T1) | |
|  | Study 1: Factory worker sample^†^ | Study 2: Flight attendant sample^‡^ |
| ***Physical health*** |  |  |
| General health | -.06 [-.19, .06] | -.37 [-.41, -.32]*** |
| Physical health | -.02 [-.14, .10] | - |
| Physically unhealthy days | - | .36 [.31, .41]*** |
| Pain-related limitations days | - | .38 [.33, .42]*** |
| Pain-related limitations | .17 [.04, .28]** | - |
| Disability days | .06 [-.07, .19] | .34 [.29, .39]*** |
| Fatigue days (past 30 days) | - | .38 [.33, .43]*** |
| Vitality days | - | -.38 [-.43, -.33]*** |
| ***Health behavior*** |  |  |
| Sleepless days | - | .22 [.17, .27]*** |
| Adequate sleep (No = 0, Yes = 1) | -.05 [-.17, .08] | - |
| Current smoking (No = 0, Yes = 1) | -.04 [-.16, .09] | - |
| ***Mental health*** |  |  |
| Mentally unhealthy days | - | .29 [.24, .34]*** |
| Depressed mood days | - | .29 [.24, .34]*** |
| Mental health | -.04 [-.16, .09] | - |
| Depressed mood | .18 [.06, .29]** | - |
| ***Psychological well-being*** |  |  |
| Life satisfaction | .03 [-.09, .15] | -.28 [-.33, -.23]*** |
| Happiness | -.04 [-.16, .09] | -.31 [-.36, -.26]*** |
| Meaning in life | .00 [-.12, .12] | -.25 [-.30, -.20]*** |
| Sense of purpose | .01 [-.11, .13] | -.21 [-.26, -.16]*** |
| ***Character strengths*** |  |  |
| Promote good | .06 [-.07, .18] | -.15 [-.20, -.09]*** |
| Delay gratification | -.03 [-.15, .09] | -.09 [-.15, -.04]** |
| ***Social well-being*** |  |  |
| Satisfying relationships | .06 [-.06, .18] | -.22 [-.27, -.17]*** |
| Content relationships | .01 [-.11, .13] | - |
| *Note*. **p* < .05, ***p* < .01, ****p* < .001. 95% confidence intervals presented in brackets. ^#^*n* = 240–264 for analyses. ^##^*n* = 1,169–1,334 for analyses. ^†^*n* = 229–264 for analyses. ^‡^*n* = 1,210–1,304 for analyses. | | |

| Supplemental Table S6  *Associations of Tertiles of Overall Suffering (T1) With Health and Well-being Outcomes (T2) in Study 1 (Factory Worker Sample)* | | | |
| --- | --- | --- | --- |
| Outcome | Tertile 1  (reference) | Tertile 2  β/OR/RR [95% CI] | Tertile 3  β/OR/RR [95% CI] |
| ***Physical health*** |  |  |  |
| General health | 0.00 | -.12 [-.41, .17] | -.26 [-.55, .02] |
| Physical health | 0.00 | -.09 [-.39, .21] | -.12 [-.41, .16] |
| Pain-related limitations^#^ | 0.00 | .12 [-.16, .39] | .44 [.16, .72]*** |
| Disability days | 0.00 | .03 [-.25, .31] | .16 [-.13, .46] |
| ***Health behavior*** |  |  |  |
| Adequate sleep^##^ | 1.00 | .97 [.78, 1.21] | 1.00 [.81, 1.24] |
| Current smoking | 1.00 | .29 [.05, 1.76] | .50 [.11, 2.22] |
| ***Mental health*** |  |  |  |
| Mental health | 0.00 | .00 [-.27, .28] | -.13 [-.41, .14] |
| Depressed mood^###^ | 0.00 | .23 [-.07, .53] | .42 [.12, .71]* |
| ***Psychological well-being*** |  |  |  |
| Life satisfaction | 0.00 | .03 [-.28, .34] | .01 [-.27, .29] |
| Happiness | 0.00 | .04 [-.24, .33] | -.00 [-.28, .28] |
| Meaning in life | 0.00 | -.12 [-.42, .18] | -.03 [-.32, .26] |
| Sense of purpose | 0.00 | -.05 [-.35, .25] | .05 [-.26, .35] |
| ***Character strengths*** |  |  |  |
| Promote good | 0.00 | .15 [-.14, .44] | .10 [-.17, .38] |
| Delay gratification | 0.00 | -.14 [-.42, .15] | -.13 [-.41, .15] |
| ***Social well-being*** |  |  |  |
| Satisfying relationships | 0.00 | .08 [-.22, .38] | .08 [-.20, .36] |
| Content relationships | 0.00 | -.00 [-.28, .28] | .05 [-.21, .32] |
| *Note*. β = standardized effect size, CI = confidence interval, OR = odds ratio, RR = risk ratio. *n* = 344 for all analyses. Multiple imputation was performed to impute missing data on the exposure, covariates, and outcomes. We ran a different type of model depending on the nature of the outcome: (1) for each continuous outcome, we ran a linear regression model to estimate a β; (2) for the outcome of current smoking (prevalence of < 10%), we ran a logistic regression model to estimate an OR; (3) for the outcome of adequate sleep (prevalence of ≥ 10%), we ran a generalized linear model with a log link and Poisson distribution to estimate a RR. If the reference value is “0,” the effect estimate is β; if the reference value is “1,” the effect estimate is OR or RR. Each continuous outcome was standardized (*M* = 0, *SD* = 1). All models adjusted for prior values of age, gender, ethnic status, marital status, educational attainment, job tenure, child dependents, and older adult dependents assessed at T1. Unless otherwise indicated, all models also adjusted for the prior value of the respective outcome assessed at T1. If a prior value of an outcome was not available, we adjusted for the prior value of a variable that was most comparable to the outcome: ^#^pain-related limitations days, ^##^sleepless days, ^###^depressed mood days (see Supplemental Table S2). **p* < .05 before Bonferroni correction, ****p* < .05 after Bonferroni correction (the *p*-value cutoff for Bonferroni correction was .05/16 = .003 for each outcome). | | | |

| Supplemental Table S7  *Complete-Case Analysis for Associations of Overall Suffering (T1) With Health and Well-being Outcomes (T2) in Study 1 (Factory Worker Sample) and Study 2 (Flight Attendant Sample)* | | | |
| --- | --- | --- | --- |
| Outcome | Reference | Study 1: Factory worker sample^#^ | Study 2: Flight attendant sample^##^ |
|  |  | β/OR/RR [95% CI] | β [95% CI] |
| ***Physical health*** |  |  |  |
| General health | 0.00 | -.03 [-.09, .03] | -0.09 [-.15, -.04]*** |
| Physical health | 0.00 | -.00 [-.06, .06] | - |
| Physically unhealthy days | 0.00 | - | .29 [.22, .36]*** |
| Pain-related limitations days | 0.00 | - | .22 [.15, .30]*** |
| Pain-related limitations^a^ | 0.00 | .09 [.03, .15]*** | - |
| Disability days | 0.00 | -.01 [-.07, .05] | .28 [.20, .35]*** |
| Fatigue days (past 30 days)^b^ | 0.00 | - | .21 [.15, .28]*** |
| Vitality days | 0.00 | - | -.21 [-.28, -.15]*** |
| ***Health behavior*** |  |  |  |
| Sleepless days | 0.00 | - | .11 [.05, .17]*** |
| Adequate sleep^c^ | 1.00 | 1.00 [.95, 1.04] | - |
| Current smoking | 1.00 | .96 [.67, 1.34] | - |
| ***Mental health*** |  |  |  |
| Mentally unhealthy days | 0.00 | - | .14 [.07, .21]*** |
| Depressed mood days^d^ | 0.00 | - | .10 [.03, .17]* |
| Mental health | 0.00 | -.01 [-.07, .05] | - |
| Depressed mood^e^ | 0.00 | .05 [-.01, .11] | - |
| ***Psychological well-being*** |  |  |  |
| Life satisfaction | 0.00 | -.00 [-.06, .06] | -.07 [-.14, -.00]* |
| Happiness | 0.00 | -.05 [-.10, .01] | -.04 [-.10, .03] |
| Meaning in life | 0.00 | .03 [-.03, .09] | -.05 [-.12, .01] |
| Sense of purpose | 0.00 | -.01 [-.07, .05] | -.01 [-.07, .05] |
| ***Character strengths*** |  |  |  |
| Promote good | 0.00 | .02 [-.04, .08] | -.11 [-.17, -.05]*** |
| Delay gratification | 0.00 | .01 [-.05, .07] | -.07 [-.13, -.00]* |
| ***Social well-being*** |  |  |  |
| Satisfying relationships | 0.00 | .01 [-.04, .07] | -.06 [-.12, .00] |
| Content relationships | 0.00 | .01 [-.04, .07] | - |
| *Note*. β = standardized effect size, CI = confidence interval, OR = odds ratio, RR = risk ratio. ^#^*n* = 144 for all analyses, ^##^*n* = 922 for all analyses. We ran a different type of model depending on the nature of the outcome: (1) for each continuous outcome, we ran a linear regression model to estimate a β; (2) for the outcome of current smoking (prevalence of < 10%), we ran a logistic regression model to estimate an OR; (3) for the outcome of adequate sleep (prevalence of ≥ 10%), we ran a generalized linear model with a log link and Poisson distribution to estimate a RR. If the reference value is “0,” the effect estimate is β; if the reference value is “1,” the effect estimate is OR or RR. Each continuous outcome was standardized (*M* = 0, *SD* = 1). All models adjusted for prior values of age, gender, sexual orientation (Study 2 only), racial status (Study 2 only), ethnic status (Study 1 only), marital status, educational attainment, job tenure, child dependents, and older adult dependents assessed at T1. Unless otherwise indicated, all models also adjusted for the prior value of the respective outcome assessed at T1. If a prior value of an outcome was not available, we adjusted for the prior value of a variable that was most comparable to the outcome: ^a^pain-related limitations days, ^b^fatigue days (past 7 days), ^c^sleepless days, ^d^depression symptoms, ^e^depressed mood days (see Supplemental Tables S2 and S10). **p* < .05 before Bonferroni correction, ****p* < .05 after Bonferroni correction (the *p*-value cutoff for Bonferroni correction was .05/16 = .003 for each outcome in both Studies 1 and 2). | | | |

| Supplemental Table S8  *Associations of Aspects of Suffering (T1) With Health and Well-being Outcomes (T2) in Study 1 (Factory Worker Sample)* | | | | | | | |
| --- | --- | --- | --- | --- | --- | --- | --- |
| Outcome | Extent of  suffering  β/OR/RR [95% CI] | Intensity of suffering  β/OR/RR [95% CI] | Length of  suffering  β/OR/RR [95% CI] | Powerlessness over suffering  β/OR/RR [95% CI] | Pervasiveness of suffering  β/OR/RR [95% CI] | Disruption to purposes  β/OR/RR [95% CI] | Threats to personhood  β/OR/RR [95% CI] |
| ***Physical health*** |  |  |  |  |  |  |  |
| General health | -.07 [-.18, .05] | -.09 [-.20, .02] | -.15 [-.27, -.03]* | -.10 [-.22, .01] | -.06 [-.18, .07] | .00 [-.11, .12] | -.11 [-.23, -.00]* |
| Physical health | -.02 [-.13, .10] | .02 [-.10, .14] | -.01 [-.12, .10] | -.08 [-.19, .04] | -.07 [-.18, .04] | -.02 [-.13, .09] | .03 [-.08, .15] |
| Pain-related limitations^#^ | .12 [.01, .24]* | .14 [.03, .26]* | .13 [.02, .25]* | .17 [.06, .28]* | .19 [.07, .30]*** | .16 [.04, .27]* | .14 [.03, .26]* |
| Disability days | .10 [-.04, .24] | .08 [-.04, .19] | .00 [-.12, .12] | .06 [-.05, .18] | .02 [-.10, .15] | .05 [-.07, .16] | .03 [-.09, .15] |
| ***Health behavior*** |  |  |  |  |  |  |  |
| Adequate sleep^##^ | .99 [.91, 1.09] | 1.00 [.92, 1.09] | 1.01 [.92, 1.10] | 1.00 [.92, 1.10] | 1.00 [.91, 1.09] | .99 [.91, 1.08] | 1.01 [.93, 1.11] |
| Current smoking | .60 [.29, 1.27] | .76 [.40, 1.44] | .92 [.47, 1.81] | .59 [.29, 1.21] | .86 [.43, 1.73] | .71 [.36, 1.41] | .59 [.29, 1.21] |
| ***Mental health*** |  |  |  |  |  |  |  |
| Mental health | -.05 [-.17, .07] | -.01 [-.13, .10] | -.01 [-.12, .10] | -.07 [-.18, .04] | -.03 [-.14, .08] | -.06 [-.17, .06] | -.03 [-.15, .08] |
| Depressed mood^###^ | .08 [-.04, .21] | .12 [-.00, .24] | .10 [-.02, .22] | .15 [.04, .26]* | .13 [.01, .25]* | .11 [-.01, .22] | .12 [-.00, .24] |
| ***Psychological well-being*** |  |  |  |  |  |  |  |
| Life satisfaction | .02 [-.10, .13] | .05 [-.06, .17] | .06 [-.05, .17] | -.04 [-.16, .07] | .05 [-.07, .16] | -.01 [-.13, .10] | .02 [-.09, .14] |
| Happiness | -.03 [-.14, .08] | .00 [-.11, .11] | -.00 [-.11, .11] | -.00 [-.11, .11] | -.05 [-.16, .06] | .01 [-.10, .12] | .07 [-.04, .19] |
| Meaning in life | -.05 [-.16, .07] | -.01 [-.12, .10] | .03 [-.08, .15] | -.03 [-.14, .09] | .00 [-.12, .12] | -.08 [-.19, .03] | .03 [-.09, .15] |
| Sense of purpose | -.04 [-.15, .07] | -.04 [-.16, .09] | -.02 [-.14, .11] | .01 [-.10, .13] | -.01 [-.13, .11] | .03 [-.09, .14] | .07 [-.04, .18] |
| ***Character strengths*** |  |  |  |  |  |  |  |
| Promote good | -.05 [-.17, .06] | .04 [-.08, .16] | .05 [-.06, .17] | .09 [-.02, .20] | .01 [-.11, .13] | .05 [-.07, .16] | .00 [-.11, .12] |
| Delay gratification | -.10 [-.22, .01] | -.11 [-.22, .00] | -.02 [-.13, .10] | -.02 [-.13, .09] | .02 [-.09, .13] | -.03 [-.15, .08] | .04 [-.07, .16] |
| ***Social well-being*** |  |  |  |  |  |  |  |
| Satisfying relationships | -.05 [-.16, .07] | .03 [-.08, .14] | .05 [-.06, .16] | .09 [-.02, .21] | .05 [-.06, .17] | -.02 [-.13, .09] | .11 [.00, .23]* |
| Content relationships | -.02 [-.13, .08] | .07 [-.04, .18] | .04 [-.07, .14] | -.00 [-.11, .11] | .07 [-.04, .18] | -.01 [-.12, .10] | .01 [-.11, .12] |
| *Note*. β = standardized effect size, CI = confidence interval, OR = odds ratio, RR = risk ratio. *n* = 344 for all analyses. Multiple imputation was performed to impute missing data on the exposure, covariates, and outcomes. For each aspect of suffering, we ran a different type of model depending on the nature of the outcome: (1) for each continuous outcome, we ran a linear regression model to estimate a β; (2) for the outcome of current smoking (prevalence of < 10%), we ran a logistic regression model to estimate an OR; (3) for the outcome of adequate sleep (prevalence of ≥ 10%), we ran a generalized linear model with a log link and Poisson distribution to estimate a RR. If the reference value is “0,” the effect estimate is β; if the reference value is “1,” the effect estimate is OR or RR. Each continuous outcome was standardized (*M* = 0, *SD* = 1). All models adjusted for prior values of age, gender, ethnic status, marital status, educational attainment, job tenure, child dependents, and older adult dependents assessed at T1. Unless otherwise indicated, all models also adjusted for the prior value of the respective outcome assessed at T1. If a prior value of an outcome was not available, we adjusted for the prior value of a variable that was most comparable to the outcome: ^#^pain-related limitations days, ^##^sleepless days, ^###^depressed mood days (see Supplemental Table S2). **p* < .05 before Bonferroni correction, ****p* < .05 after Bonferroni correction (the *p*-value cutoff for Bonferroni correction was .05/16 = .003 for each outcome). | | | | | | | |

| Supplemental Table S9  *Baseline Characteristics of Participants in Study 2 (Flight Attendant Sample) Who Completed Both T1 and T2 Surveys and Those Who Did Not* | | | | | | | | | | | |
| --- | --- | --- | --- | --- | --- | --- | --- | --- | --- | --- | --- |
| Characteristic | Total (*n* = 7,338) | | |  | T1 and T2 (*n* = 1,402) | |  | T1 only (*n* = 5,936) | | *p*-value | *d* |
|  | *n* | % | *M* ± *SD* (range) |  | % | *M* ± *SD* (range) |  | % | *M* ± *SD* (range) |  |  |
| **Sociodemographics** |  |  |  |  |  |  |  |  |  |  |  |
| Age (years) | 7,044 |  | 47.63 ± 12.26 (18–78) |  |  | 56.09 ± 6.10 (46–75) |  |  | 45.54 ± 12.51 (18–78) | < .001 | .92 |
| Gender | 7,332 |  |  |  |  |  |  |  |  | .003 | .07 |
| Female^a^ | 5,828 | 79.49 |  |  | 82.38 |  |  | 78.80 |  |  |  |
| Male | 1,504 | 20.51 |  |  | 17.62 |  |  | 21.20 |  |  |  |
| Sexual orientation | 7,294 |  |  |  |  |  |  |  |  | < .001 | .12 |
| Heterosexual | 6,132 | 84.07 |  |  | 88.56 |  |  | 83.01 |  |  |  |
| Other | 1,162 | 15.93 |  |  | 11.44 |  |  | 16.99 |  |  |  |
| Racial status | 7,306 |  |  |  |  |  |  |  |  | < .001 | .09 |
| White | 5,890 | 80.62 |  |  | 84.42 |  |  | 79.72 |  |  |  |
| Other | 1,416 | 19.38 |  |  | 15.58 |  |  | 20.28 |  |  |  |
| Marital status | 5,558 |  |  |  |  |  |  |  |  | < .001 | .16 |
| Married | 2,526 | 45.45 |  |  | 52.83 |  |  | 43.33 |  |  |  |
| Other | 3,032 | 54.55 |  |  | 47.17 |  |  | 56.67 |  |  |  |
| Educational attainment | 5,558 |  |  |  |  |  |  |  |  | .264 | .03 |
| Up to completion of undergraduate degree | 4,958 | 89.20 |  |  | 88.30 |  |  | 89.47 |  |  |  |
| Graduate school/advanced degree | 600 | 10.80 |  |  | 11.70 |  |  | 10.53 |  |  |  |
| Child dependents | 5,429 |  |  |  |  |  |  |  |  | < .001 | .11 |
| No | 4,239 | 78.08 |  |  | 82.24 |  |  | 76.89 |  |  |  |
| Yes | 1,190 | 21.92 |  |  | 17.76 |  |  | 23.11 |  |  |  |
| Older adult dependents | 5,540 |  |  |  |  |  |  |  |  | .009 | .07 |
| No | 4,849 | 87.53 |  |  | 85.33 |  |  | 88.16 |  |  |  |
| Yes | 691 | 12.47 |  |  | 14.67 |  |  | 11.84 |  |  |  |
| Employment tenure | 7,237 |  |  |  |  |  |  |  |  | < .001 | .12 |
| ≤ 5 years | 6,696 | 92.52 |  |  | 95.77 |  |  | 91.75 |  |  |  |
| > 5 years | 541 | 7.48 |  |  | 4.23 |  |  | 8.25 |  |  |  |
| **Suffering** |  |  |  |  |  |  |  |  |  |  |  |
| Overall suffering | 6,560 |  | 2.40 ± 2.53 (0–10) |  |  | 2.21 ± 2.41 (0–9.71) |  |  | 2.45 ± 2.55 (0–10) | .001 | .09 |
| **Physical health** |  |  |  |  |  |  |  |  |  |  |  |
| General health | 7,075 |  | 3.57 ± 0.92 (1–5) |  |  | 3.67 ± 0.92 (1–5) |  |  | 3.54 ± 0.91 (1–5) | < .001 | .15 |
| Physically unhealthy days | 6,919 |  | 5.15 ± 7.82 (0–30) |  |  | 5.12 ± 7.90 (0–30) |  |  | 5.16 ± 7.81 (0–30) | .882 | .00 |
| Pain-related limitations days | 6,786 |  | 5.22 ± 8.33 (0–30) |  |  | 5.88 ± 8.87 (0–30) |  |  | 5.06 ± 8.19 (0–30) | .002 | .10 |
| Disability days | 6,756 |  | 4.27 ± 7.20 (0–30) |  |  | 4.08 ± 7.33 (0–30) |  |  | 4.32 ± 7.17 (0–30) | .284 | .03 |
| Fatigue days (past 7 days) | 6,171 |  | 2.54 ± 1.35 (1–5) |  |  | 2.48 ± 1.35 (1–5) |  |  | 2.55 ± 1.35 (1–5) | .075 | .06 |
| Vitality days | 5,399 |  | 11.60 ± 9.17 (0–30) |  |  | 12.53 ± 9.52 (0–30) |  |  | 11.34 ± 9.05 (0–30) | < .001 | .13 |
| **Health behavior** |  |  |  |  |  |  |  |  |  |  |  |
| Sleepless days | 5,418 |  | 11.73 ± 8.61 (0–30) |  |  | 11.61 ± 8.82 (0–30) |  |  | 11.76 ± 8.55 (0–30) | .588 | .02 |
| **Mental health** |  |  |  |  |  |  |  |  |  |  |  |
| Mentally unhealthy days | 6,884 |  | 5.85 ± 7.84 (0–30) |  |  | 5.13 ± 7.49 (0–30) |  |  | 6.02 ± 7.92 (0–30) | < .001 | .11 |
| Depression symptoms | 6,107 |  | 6.80 ± 5.27 (0–27) |  |  | 5.79 ± 4.80 (0–26) |  |  | 7.06 ± 5.35 (0–27) | < .001 | .24 |
| **Psychological well-being** |  |  |  |  |  |  |  |  |  |  |  |
| Life satisfaction | 6,449 |  | 7.04 ± 2.02 (0–10) |  |  | 7.26 ± 1.97 (0–10) |  |  | 6.98 ± 2.04 (0–10) | < .001 | .14 |
| Happiness | 6,450 |  | 7.06 ± 1.87 (0–10) |  |  | 7.31 ± 1.75 (0–10) |  |  | 6.99 ± 1.89 (0–10) | < .001 | .17 |
| Meaning in life | 6,483 |  | 7.46 ± 2.05 (0–10) |  |  | 7.69 ± 1.90 (0–10) |  |  | 7.40 ± 2.08 (0–10) | < .001 | .14 |
| Sense of purpose | 6,444 |  | 6.87 ± 2.44 (0–10) |  |  | 7.23 ± 2.26 (0–10) |  |  | 6.77 ± 2.48 (0–10) | < .001 | .19 |
| **Character strengths** |  |  |  |  |  |  |  |  |  |  |  |
| Promote good | 6,447 |  | 7.91 ± 1.65 (0–10) |  |  | 8.05 ± 1.53 (0–10) |  |  | 7.87 ± 1.67 (0–10) | < .001 | .11 |
| Delay gratification | 6,360 |  | 7.19 ± 1.92 (0–10) |  |  | 7.38 ± 1.85 (0–10) |  |  | 7.14 ± 1.94 (0–10) | < .001 | .13 |
| **Social well-being** |  |  |  |  |  |  |  |  |  |  |  |
| Satisfying relationships | 6,377 |  | 6.29 ± 2.61 (0–10) |  |  | 6.50 ± 2.51 (0–10) |  |  | 6.24 ± 2.63 (0–10) | < .001 | .10 |
| *Note*. *d* = Cohen’s *d*, *M* = mean, *SD* = standard deviation. Percentages refer to the proportion of individuals within each inclusion category with that characteristic. *p*-values come from independent samples *t*-tests or *χ*^2^ tests that were used to examine the mean levels of the characteristic or the proportion of individuals within the retained and lost to follow-up categories with that characteristic. ^a^Includes three participants who identified as transgender but only responded to the T1 survey. | | | | | | | | | | | |

| Supplemental Table S10  *List of Items Used for Exposure and Outcome Variables in Study 2 (Flight Attendant Sample)* | | | |
| --- | --- | --- | --- |
| Variable | Item/question | Response scale | Assessment |
| **Suffering**^1^ |  |  | T1 |
| Extent of suffering | To what extent are you suffering? | 0 (*Not suffering at all*) to 10 (*Suffering terribly*) |  |
| Intensity of suffering | The intensity of my suffering feels intolerable. | 0 (*Do not agree*) to 10 (*Completely agree*) |  |
| Length of suffering | The length of time I have been suffering or expect to suffer, feels intolerable. | 0 (*Do not agree*) to 10 (*Completely agree*) |  |
| Powerlessness over suffering | I feel powerless to stop my current suffering. | 0 (*Do not agree*) to 10 (*Completely agree*) |  |
| Pervasiveness of suffering | The suffering I have been experiencing affects all aspects of my life. | 0 (*Do not agree*) to 10 (*Completely agree*) |  |
| Disruption to purposes | Certain purposes of my life have been badly disrupted because of my suffering. | 0 (*Do not agree*) to 10 (*Completely agree*) |  |
| Threats to personhood | My experience of suffering threatens who I am as a person. | 0 (*Do not agree*) to 10 (*Completely agree*) |  |
| **Physical health** |  |  |  |
| General health^2,3^ | Would you say that in general your health is… | 1 (*Poor*) to 5 (*Excellent*) | T1 & T2 |
| Physically unhealthy days^2,3^ | Now thinking about your physical health, which includes physical illness and injury, for how many days during the past 30 days was your physical health not good? | 0 to 30 (*Days*) | T1 & T2 |
| Pain-related limitations days^2,3^ | During the past 30 days, for about how many days did pain make it hard for you to do your usual activities, such as self-care, work, or recreation? | 0 to 30 (*Days*) | T1 & T2 |
| Disability days^2,3^ | During the past 30 days, for about how many days did poor physical or mental health keep you from doing your usual activities, such as self-care, work, or recreation? | 0 to 30 (*Days*) | T1 & T2 |
| Fatigue days (past 7 days) | In the past 7 days, how many days did you experience the following symptoms? - Unusual tiredness or fatigue | 1 (*Never/0 days*) to 5 (*Everyday/7 days*) | T1 |
| Fatigue days (past 30 days) | In the past 30 DAYS, how often did you experience the following symptoms? - Fatigue | 1 (*Never*) to 5 (*Everyday*) | T2 |
| Vitality days^2,3^ | During the past 30 days, how many days did you feel very healthy and full of energy? | 0 to 30 (*Days*) | T1 & T2 |
| **Health behavior** |  |  |  |
| Sleepless days^2,3^ | During the past 30 days, how many days have you NOT gotten enough rest or sleep? | 0 to 30 (*Days*) | T1 & T2 |
| **Mental health** |  |  |  |
| Mentally unhealthy days^2,3^ | Now thinking about your mental health, which includes stress, depression, and problems with emotions, for how many days during the past 30 days was your mental health not good? | 0 to 30 (*Days*) | T1 & T2 |
| Depression symptoms^6^ |  |  | T1 |
| PHQ1 | Little interest or pleasure in doing things? | 0 (*Not at all*) to 3 (*Nearly every day*) |  |
| PHQ2 | Feeling down, depressed, or hopeless? | 0 (*Not at all*) to 3 (*Nearly every day*) |  |
| PHQ3 | Trouble falling or staying asleep, or sleeping too much? | 0 (*Not at all*) to 3 (*Nearly every day*) |  |
| PHQ4 | Feeling tired or having little energy? | 0 (*Not at all*) to 3 (*Nearly every day*) |  |
| PHQ5 | Poor appetite or overeating? | 0 (*Not at all*) to 3 (*Nearly every day*) |  |
| PHQ6 | Feeling bad about yourself- that you are a failure and have let yourself or your family down? | 0 (*Not at all*) to 3 (*Nearly every day*) |  |
| PHQ7 | Trouble concentrating on things, such as reading the newspaper or watching TV? | 0 (*Not at all*) to 3 (*Nearly every day*) |  |
| PHQ8 | Moving or speaking so slowly that other people could have noticed? Or the opposite – being so fidgety or restless that you have been moving around a lot more than usual? | 0 (*Not at all*) to 3 (*Nearly every day*) |  |
| PHQ9 | Thoughts that you would be better off dead or of hurting yourself in some way? | 0 (*Not at all*) to 3 (*Nearly every day*) |  |
| Depressed mood days^2,3^ | During the past 30 days, for about how many days did you feel sad or depressed? | 0 to 30 (*Days*) | T2 |
| **Psychological well-being** |  |  |  |
| Life satisfaction^4^ | Overall, how satisfied are you with life as a whole these days? | 0 (*Not at all satisfied*) to 10 (*Completely satisfied*) | T1 & T2 |
| Happiness^4^ | In general, how happy or unhappy do you usually feel? | 0 (*Extremely unhappy*) to 10 (*Extremely happy*) | T1 & T2 |
| Meaning in life^4^ | Overall, to what extent do you feel the things you do in your life are worthwhile? | 0 (*Not at all worthwhile*) to 10 (*Completely worthwhile*) | T1 & T2 |
| Sense of purpose^4^ | I understand my purpose in life. | 0 (*Strongly disagree*) 10 (*Strongly agree*) | T1 & T2 |
| **Character strengths** |  |  |  |
| Promote good^4^ | I always act to promote good in all circumstances, even in difficult and challenging situations. | 0 (*Strongly disagree*) 10 (*Strongly agree*) | T1 & T2 |
| Delay gratification^4^ | I am always able to give up some happiness now for greater happiness later. | 0 (*Strongly disagree*) 10 (*Strongly agree*) | T1 & T2 |
| **Social well-being** |  |  |  |
| Satisfying relationships^4^ | My relationships are as satisfying as I would want them to be. | 0 (*Strongly disagree*) 10 (*Strongly agree*) | T1 & T2 |
| *Note*. Personal Suffering Assessment^1^; Centers for Disease Control and Prevention Health-Related Quality of Life 14^2,3^; Flourishing Index^4^; Patient Health Questionnaire-9^6^. | | | |

| Supplemental Table S11  *Associations of Tertiles of Overall Suffering (T1) With Health and Well-being Outcomes (T2) in Study 2 (Flight Attendant Sample)* | | | |
| --- | --- | --- | --- |
| Outcome | Tertile 1  (reference) | Tertile 2  β [95% CI] | Tertile 3  β [95% CI] |
| ***Physical health*** |  |  |  |
| General health | 0.00 | -.12 [-.24, -.01]* | -.20 [-.33, -.07]*** |
| Physically unhealthy days | 0.00 | .15 [.02, .29]* | .62 [.47, .77]*** |
| Pain-related limitations days | 0.00 | .19 [.06, .32]* | .47 [.31, .63]*** |
| Disability days | 0.00 | .15 [.02, .29]* | .55 [.40, .71]*** |
| Fatigue days (past 30 days)^#^ | 0.00 | .28 [.15, .42]*** | .46 [.31, .61]*** |
| Vitality days | 0.00 | -.27 [-.40, -.14]*** | -.54 [-.69, -.39]*** |
| ***Health behavior*** |  |  |  |
| Sleepless days | 0.00 | .16 [.04, .29]* | .34 [.21, .47]*** |
| ***Mental health*** |  |  |  |
| Mentally unhealthy days | 0.00 | .14 [.01, .27]* | .32 [.17, .46]*** |
| Depressed mood days^##^ | 0.00 | .02 [-.11, .15] | .20 [.05, .35]* |
| ***Psychological well-being*** |  |  |  |
| Life satisfaction | 0.00 | -.09 [-.22, .04] | -.14 [-.29, .00] |
| Happiness | 0.00 | -.05 [-.17, .06] | -.12 [-.25, .02] |
| Meaning in life | 0.00 | -.04 [-.17, .09] | -.09 [-.22, .05] |
| Sense of purpose | 0.00 | .02 [-.09, .14] | -.02 [-.14, .10] |
| ***Character strengths*** |  |  |  |
| Promote good | 0.00 | -.07 [-.19, .05] | -.18 [-.30, -.05]* |
| Delay gratification | 0.00 | -.13 [-.26, .01] | -.05 [-.18, .09] |
| ***Social well-being*** |  |  |  |
| Satisfying relationships | 0.00 | .04 [-.08, .17] | -.09 [-.23, .04] |
| *Note*. β = standardized effect size, CI = confidence interval. *n* = 1,402 for all analyses. Multiple imputation was performed to impute missing data on the exposure, covariates, and outcomes. We ran a linear regression model to estimate a β for all outcomes (one outcome at a time). Each outcome was continuous and standardized (*M* = 0, *SD* = 1). All models adjusted for prior values of age, gender, sexual orientation, racial status, marital status, educational attainment, job tenure, child dependents, and older adult dependents assessed at T1. Unless otherwise indicated, all models also adjusted for the prior value of the respective outcome assessed at T1. If a prior value of an outcome was not available, we adjusted for the prior value of a variable that was most comparable to the outcome: ^#^fatigue days (past 7 days), ^##^depression symptoms (see Supplemental Table S10). **p* < .05 before Bonferroni correction, ****p* < .05 after Bonferroni correction (the *p*-value cutoff for Bonferroni correction was .05/16 = .003 for each outcome). | | | |

| Supplemental Table S12  *Associations of Aspects of Suffering (T1) With Health and Well-being Outcomes (T2) in Study 2 (Flight Attendant Sample)* | | | | | | | |
| --- | --- | --- | --- | --- | --- | --- | --- |
| Outcome | Extent of  suffering  β [95% CI] | Intensity of  suffering  β [95% CI] | Length of  suffering  β [95% CI] | Powerlessness over suffering  β [95% CI] | Pervasiveness of suffering  β [95% CI] | Disruption to purposes  β [95% CI] | Threats to personhood  β [95% CI] |
| ***Physical health*** |  |  |  |  |  |  |  |
| General health | -.10 [-.16, -.05]*** | -.08 [-.13, -.03]*** | -.05 [-.10, .00] | -.06 [-.11, -.01]* | -.07 [-.12, -.02]* | -.08 [-.13, -.03]*** | -.06 [-.11, -.01]* |
| Physically unhealthy days | .29 [.22, .35]*** | .21 [.15, .27]*** | .23 [.16, .29]*** | .26 [.20, .32]*** | .28 [.22, .34]*** | .28 [.22, .34]*** | .24 [.18, .30]*** |
| Pain-related limitations days | .17 [.10, .24]*** | .14 [.08, .20]*** | .16 [.10, .22]*** | .20 [.14, .26]*** | .23 [.17, .30]*** | .22 [.16, .29]*** | .19 [.13, .25]*** |
| Disability days | .19 [.12, .26]*** | .15 [.08, .21]*** | .14 [.08, .21]*** | .22 [.15, .28]*** | .24 [.17, .30]*** | .29 [.23, .36]*** | .28 [.22, .35]*** |
| Fatigue days (past 30 days)^#^ | .18 [.12, .24]*** | .12 [.06, .18]*** | .14 [.08, .20]*** | .16 [.10, .22]*** | .20 [.14, .26]*** | .20 [.14, .25]*** | .14 [.09, .20]*** |
| Vitality days | -.20 [-.26, -.14]*** | -.14 [-.20, -.08]*** | -.16 [-.21, -.10]*** | -.20 [-.26, -.14]*** | -.19 [-.25, -.13]*** | -.22 [-.27, -.16]*** | -.18 [-.24, -.13]*** |
| ***Health behavior*** |  |  |  |  |  |  |  |
| Sleepless days | .11 [.06, .16]*** | .06 [.00, .11]* | .08 [.02, .13]* | .10 [.05, .16]*** | .10 [.05, .16]*** | .11 [.06, .17]*** | .09 [.04, .14]*** |
| ***Mental health*** |  |  |  |  |  |  |  |
| Mentally unhealthy days | .08 [.02, .14]* | .08 [.02, .14]* | .07 [.01, .13]* | .11 [.05, .17]*** | .12 [.06, .18]*** | .12 [.06, .18]*** | .16 [.09, .22]*** |
| Depressed mood days^##^ | .05 [-.01, .11] | .06 [.00, .12]* | .05 [-.00, .11] | .09 [.03, .15]* | .09 [.02, .15]* | .09 [.03, .15]* | .16 [.10, .22]*** |
| ***Psychological well-being*** |  |  |  |  |  |  |  |
| Life satisfaction | -.05 [-.10, .01] | -.05 [-.11, .00] | -.07 [-.13, -.01]* | -.06 [-.11, .00] | -.08 [-.14, -.02]* | -.08 [-.14, -.03]* | -.09 [-.15, -.02]* |
| Happiness | -.03 [-.08, .02] | -.06 [-.11, -.01]* | -.04 [-.09, .02] | -.06 [-.11, -.01]* | -.05 [-.11, -.00]* | -.06 [-.11, -.01]* | -.08 [-.13, -.02]* |
| Meaning in life | -.06 [-.11, -.01]* | -.10 [-.15, -.04]*** | -.07 [-.13, -.02]* | -.08 [-.13, -.02]* | -.07 [-.13, -.02]* | -.08 [-.13, -.02]* | -.10 [-.16, -.05]*** |
| Sense of purpose | -.02 [-.07, .03] | -.04 [-.09, .01] | -.03 [-.08, .02] | -.03 [-.08, .02] | -.04 [-.09, .01] | -.04 [-.09, .01] | -.05 [-.10, -.00]* |
| ***Character strengths*** |  |  |  |  |  |  |  |
| Promote good | -.07 [-.12, -.02]* | -.10 [-.15, -.05]*** | -.08 [-.13, -.03]* | -.09 [-.14, -.04]*** | -.07 [-.12, -.02]* | -.07 [-.12, -.02]* | -.10 [-.15, -.05]*** |
| Delay gratification | -.02 [-.07, .04] | -.02 [-.07, .04] | -.02 [-.07, .04] | -.03 [-.08, .03] | -.04 [-.10, .01] | -.01 [-.07, .04] | -.05 [-.10, .00] |
| ***Social well-being*** |  |  |  |  |  |  |  |
| Satisfying relationships | -.06 [-.11, -.00]* | -.06 [-.11, -.00]* | -.03 [-.09, .02] | -.07 [-.13, -.02]* | -.07 [-.12, -.02]* | -.07 [-.13, -.02]* | -.09 [-.15, -.04]*** |
| *Note*. β = standardized effect size, CI = confidence interval. *n* = 1,402 for all analyses. Multiple imputation was performed to impute missing data on the exposure, covariates, and outcomes. For each aspect of suffering, we ran a linear regression model to estimate a β for all outcomes (one outcome at a time). Each outcome was continuous and standardized (*M* = 0, *SD* = 1). All models adjusted for prior values of age, gender, sexual orientation, racial status, marital status, educational attainment, job tenure, child dependents, and older adult dependents assessed at T1. Unless otherwise indicated, all models also adjusted for the prior value of the respective outcome assessed at T1. If a prior value of an outcome was not available, we adjusted for the prior value of a variable that was most comparable to the outcome: ^#^fatigue days (past 7 days), ^##^depression symptoms (see Supplemental Table S10). **p* < .05 before Bonferroni correction, ****p* < .05 after Bonferroni correction (the *p*-value cutoff for Bonferroni correction was .05/16 = .003 for each outcome). | | | | | | | |

References

1. VanderWeele, T. J. Suffering and response: Directions in empirical research. *Soc. Sci. Med.* **224**, 58–66 (2019).

2. Moriarty, D. G., Zack, M. M. & Kobau, R. The Centers for Disease Control and Prevention’s Healthy Days Measures – Population tracking of perceived physical and mental health over time. *Health Qual. Life Outcomes* **1**, 37 (2003).

3. Centers for Disease Control and Prevention. *Measuring healthy days: Population assessment of health-related quality of life*. https://stacks.cdc.gov/view/cdc/6406 (2000).

4. VanderWeele, T. J. On the promotion of human flourishing. *Proc. Natl. Acad. Sci.* **114**, 8148–8156 (2017).

5. Węziak-Białowolska, D. *et al.* Psychometric properties of flourishing scales from a comprehensive well-being assessment. *Front. Psychol.* **12**, 652209 (2021).

6. Kroenke, K., Spitzer, R. L. & Williams, J. B. The PHQ-9: Validity of a brief depression severity measure. *J. Gen. Intern. Med.* **16**, 606–613 (2001).
